# Design, Synthesis, and Biological Evaluation of Efflux-Resistant Imatinib Derivatives

**DOI:** 10.1021/acs.jmedchem.5c01596

**Published:** 2025-10-25

**Authors:** Madiha M. Chowdhury, Priantha Pretheshan, Nasima S. Chowdhury, Paolo Andriollo, Ajit J. Shah, Ben Forbes, Chris Pepper, Khondaker Miraz Rahman

**Affiliations:** 1 Institute of Pharmaceutical Science, 4616 King’s College London, London SE1 9NH, U.K.; 2 Department of Natural Sciences, 4907 University of Middlesex, The Burroughs, Hendon, London NW4 4BT, U.K.; 3 Brighton and Sussex Medical School, University of Brighton and University of Sussex, Brighton BN1 9PX, U.K.

## Abstract

Resistance to imatinib, a first-line BCR-ABL1 tyrosine kinase inhibitor for chronic myeloid leukemia, is frequently mediated by drug efflux through P-glycoprotein (P-gp) overexpression. We report the design, synthesis, and evaluation of eight novel imatinib derivatives modified at the piperazine terminus with efflux resistance breaker (ERB) fragments to reduce P-gp-mediated efflux. *In silico* docking against cryo-EM P-gp structures predicted increased hydrophobic interactions and enhanced occupancy at the access tunnel, indicative of efflux inhibition. Compound **8** showed potency comparable to imatinib in BCR-ABL1^+^ K562 cells and a lower LC_50_ fold change in resistant K562/DOX cells, suggesting reduced efflux susceptibility. Accumulation assays confirmed the improved intracellular retention of compound **8**. Compound **9** displayed increased potency in resistant cells, correlating with higher intracellular levels despite modest kinase inhibition. Verapamil assays confirmed reduced efflux liability for compounds **8** and **13**. Compound **8** also showed a positive therapeutic index. These findings support rational design to mitigate efflux-mediated resistance.

## Graphical abstract

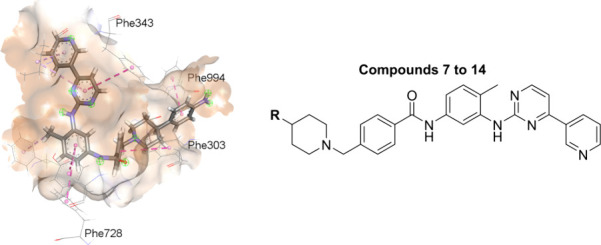

## Introduction

Chronic myeloid leukemia (CML) is characterized by the BCR-ABL1 fusion gene, which encodes a constitutively active tyrosine kinase that drives tumor cell proliferation.^1^ Imatinib, a selective BCR-ABL1 inhibitor, has transformed CML treatment and remains the first-line therapy in many settings.^2^ However, the development of resistance, both BCR-ABL1-dependent (e.g., kinase domain mutations) and independent, limits long-term efficacy.^3^ A frequently observed resistance mechanism is the overexpression of P-glycoprotein (P-gp; also known as ABCB1/MDR1), an ATP-binding cassette (ABC) transporter that reduces intracellular drug concentrations by active efflux.^4,5^

While second-generation tyrosine kinase inhibitors (TKIs) like nilotinib improve potency,^6^ many remain P-gp substrates, limiting their efficacy in resistant disease.^6^ Co-administration of efflux pump inhibitors has been explored to overcome this limitation but has failed clinically due to toxicity and poor selectivity.^8^ An alternative strategy involves direct structural modification of therapeutic agents to reduce their interaction with efflux transporters while retaining target specificity.^9^ If successful, this would address an unmet clinical need as efflux transporters such as P-gp are frequently upregulated in various cancers and contribute to multidrug resistance (MDR) by limiting intracellular drug accumulation.^10,11^ Their expression is often associated with poor prognosis, reduced treatment response, and increased likelihood of relapse, in hematological malignancies and solid tumors.^12,13^ Targeting these efflux systems is therefore an attractive strategy for restoring drug sensitivity and improving therapeutic outcomes. Notably, P-gp not only reduces drug efficacy through direct efflux but also alters intracellular drug pharmacokinetics, potentially undermining dose–response relationships and therapeutic index.^14^ So, modifying drug molecules to reduce their efflux liability or inhibit these transporters without affecting on-target activity represents a promising solution to one of the key barriers in contemporary anticancer drug development.^15^

Our team previously observed that efflux pump inhibitors tend to bind to more hydrophobic pockets within transporter structures, while substrates engage more hydrophilic regions.^16^ This was supported by computational modeling and later confirmed by several structural studies including the X-ray structure of the P-gp inhibitor encequidar bound to P-gp.^17^ Based on this, we developed a proprietary ERB technology. ERBs are chemical fragments designed to promote interactions with inhibitor-associated hydrophobic pockets of P-gp, reducing substrate-like behavior. This approach has shown promise in antibacterial and antifungal drug classes^16,18^ and is now being applied to anticancer scaffolds.

In this study, we report the first application of the ERB technology to anticancer scaffolds with imatinib as the prototype molecule due to its known efflux liability. We designed eight ERB-modified derivatives by attaching efflux-resistant fragments at the piperazine terminus of the imatinib scaffold. These compounds were evaluated for molecular binding, kinase inhibition, cytotoxicity, and efflux modulation in P-gp-overexpressing cells. Compound **8** emerged as a lead candidate with improved efficacy and reduced susceptibility to efflux, supporting the use of ERB modification to overcome transporter-mediated resistance in CML.

## Results and Discussion

### Rational Design Using Molecular Modeling

Our strategy for modifying imatinib analogs was informed by established structure–activity relationship (SAR) data,^19,20^ enabling a rational approach to retain BCR-ABL1 inhibition while addressing resistance (Figure 1). Core pharmacophores such as the PAP (Phenyl amino pyrimidine) moiety (rings C and D), pyridyl group, and *N*-methyl piperazine confer target engagement, cellular activity, and bioavailability, respectively.^19^ The benzene ring reduces mutagenicity, while modifications on ring B and ring C contribute to kinase inhibition and selectivity. To overcome P-gp-mediated drug efflux, we introduced ERB fragments at the piperazine terminus, a modification site supported by prior SAR work. These ERBs, derived from bacterial efflux transporter studies,^16,21^ included nitrogen-containing five- and six-membered heterocycles designed to interfere with P-gp substrate recognition.

**Figure 1:**
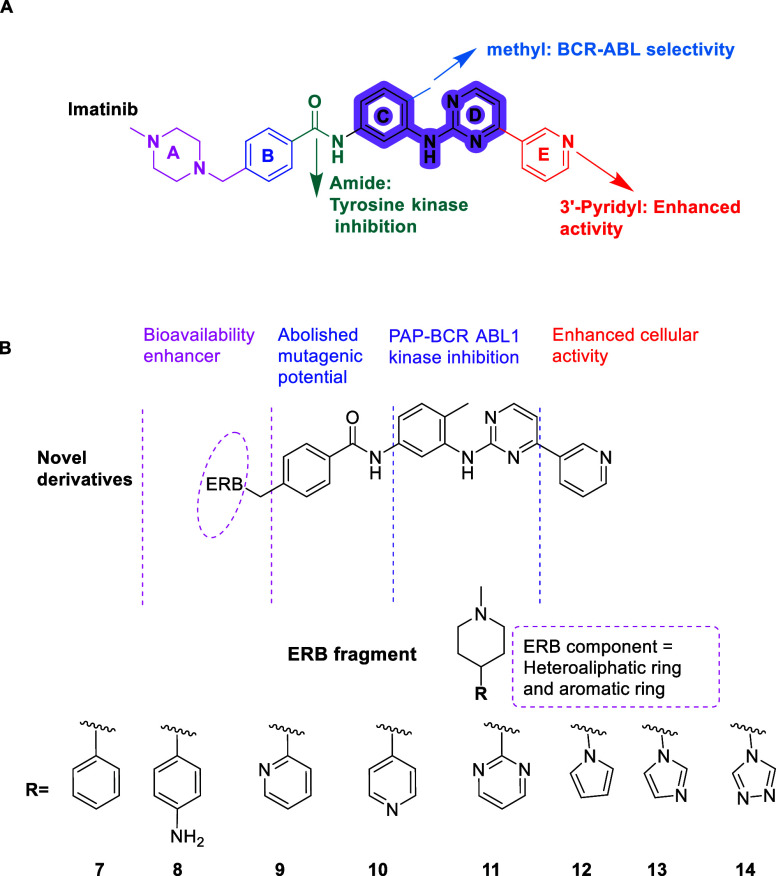
Schema showing the key functional components of imatinib and the structural adaptations introduced to create the novel compound library. A) Role of different structural components of imatinib in biological activity. B) Design strategy for target compounds. Imatinib derivatives with addition of efflux resistance breaker (ERB) fragments, composed of the *N*-methyl piperazine and the aromatic/heteroaromatic groups denoted by R.

Docking studies using a cryo-EM-based model of P-gp bound to encequidar (PDB ID: 7O9W) supported the design (Figure 2). The structure shows two encequidar molecules bound per pump, one in the substrate pocket and the other extending into the access tunnel, thereby blocking the conformational change required for efflux. The model was built to include both binding sites, which enabled assessment of analog affinity for each motif. Comparisons were made between the contacting residues found in the docked structures and the cocrystallized encequidar poses to compare location of binding and therefore inhibitor-like properties. Analogs were also evaluated for hydrophobic interactions with the pump, consistent with our previous work.^16^ As hypothesized, the analogs, particularly compound **8**, showed increased hydrophobic interactions at the P-gp access tunnel, overlapping with the inhibitory binding mode of encequidar.^17,22^ Compound **8** formed 13 interactions in the inhibitor site versus 9 in the substrate binding site, suggesting a preference for the inhibitory domain and potentially contributing to reduced efflux and enhanced intracellular retention. Furthermore, the ERB modification led to a change in the orientation of the compound within the pump. In compound **8**, the ERB fragment extends further into the access tunnel and is stabilized by pi-alkyl interactions, whereas the equivalent *N*-methylpiperidine ring in imatinib is located closer to the substrate binding pocket and is held only by weaker van der Waals forces. The overlap in contacting residues with the central core rings C and D provides further evidence that the rational modifications altered imatinib’s interactions in a way that makes it less susceptible to efflux.

**Figure 2:**
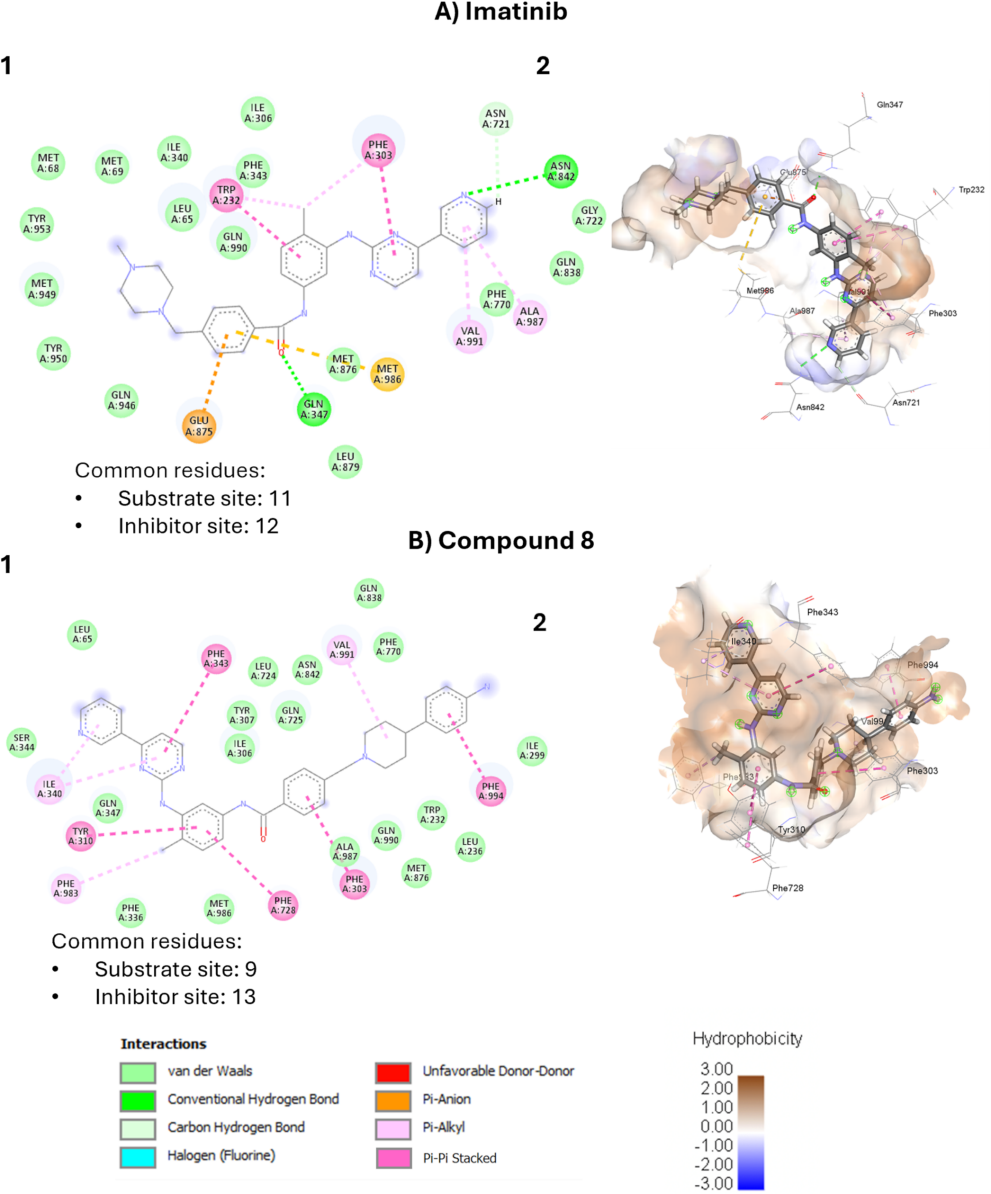
Molecular interaction poses of imatinib (A) and compound **8** (B) with P-gp inhibitory pocket. Molecular docking of most favorable poses in terms of the binding energy and commonality in access tunnel contact residues compared to encequidar, for imatinib and the compound **8** highlighting the increase in hydrophobic interactions after the addition of ERB fragments. 1) 2D-interaction diagram with human P-gp 7O9W. 2) 3D-molecular interaction where the blue region indicates hydrophilicity and brown color represents hydrophobic regions.

### Synthesis of the Compounds

ERB-modified imatinib derivatives (compounds **7**-**14**) were synthesized via a concise three-step route beginning with 4-methylbromo benzoic acid (Scheme 1). Acyl chloride formation using thionyl chloride was followed by amide coupling with substituted anilines (Scheme 2). The final S_N_2 substitution with diverse ERB amines afforded the target analogs in moderate to high yields.^23^ This synthetic route was modular and adaptable, allowing the late-stage introduction of chemically distinct ERB groups while maintaining the imatinib scaffold. This flexibility enabled systematic variation of ring size, heteroatom position, and electronic features to evaluate their influence on biological activity. All compounds were characterized by ^1^H and ^13^C NMR and ESI-MS, with purities exceeding 95% (Table S3). Where necessary, Buchwald–Hartwig coupling facilitated access to noncommercial ERB amines, and this synthetic efficiency supported the construction of a chemically diverse and pharmacologically relevant compound library (Scheme 3).

**Scheme 1:**
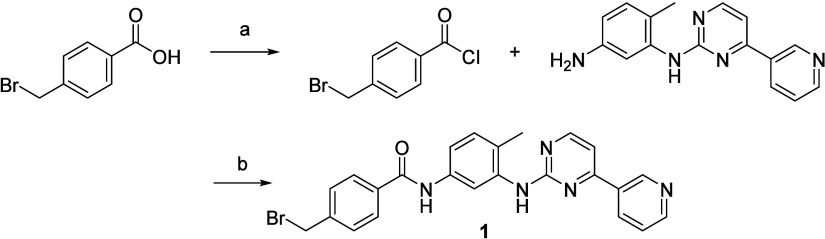
Synthesis of Key Core Intermediate^a^

a Reagents and conditions: a) Thionyl Chloride, Ar, 75°C, reflux 6 h; b) Triethylamine (TEA) 0°C, Chloroform, N_2_, reflux 10 h.

**Scheme 2:**
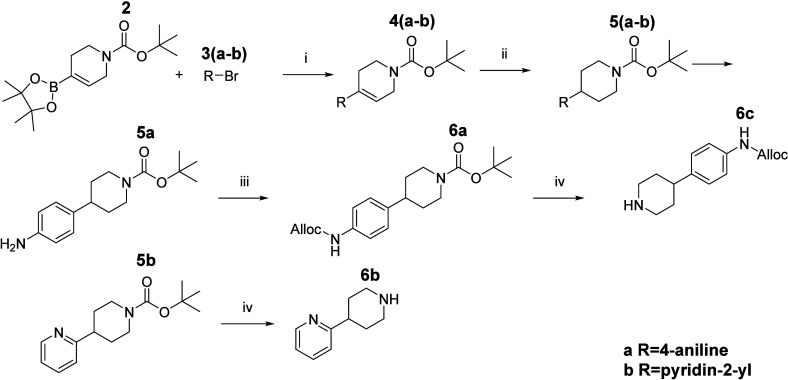
Synthesis of Commercially Unavailable ERB Tertiary Amines^a^

a Reagents and conditions: (i) Pd (PPh_3_)_4_, Na_2_CO_3_, 100 °C, 10 Pka, 50 min, N_2_, 6:2:1 tetrahydrofuran/methanol/water; (ii) H_2_, 10% w/w Pd/C, 50 psi; (iii) Dry pyridine, allyl chloroformate, dry DCM −10 °C, 2 h; (iv) 4 N HCl in dioxane, methanol, r.t.p, 2 h.

**Scheme 3:**
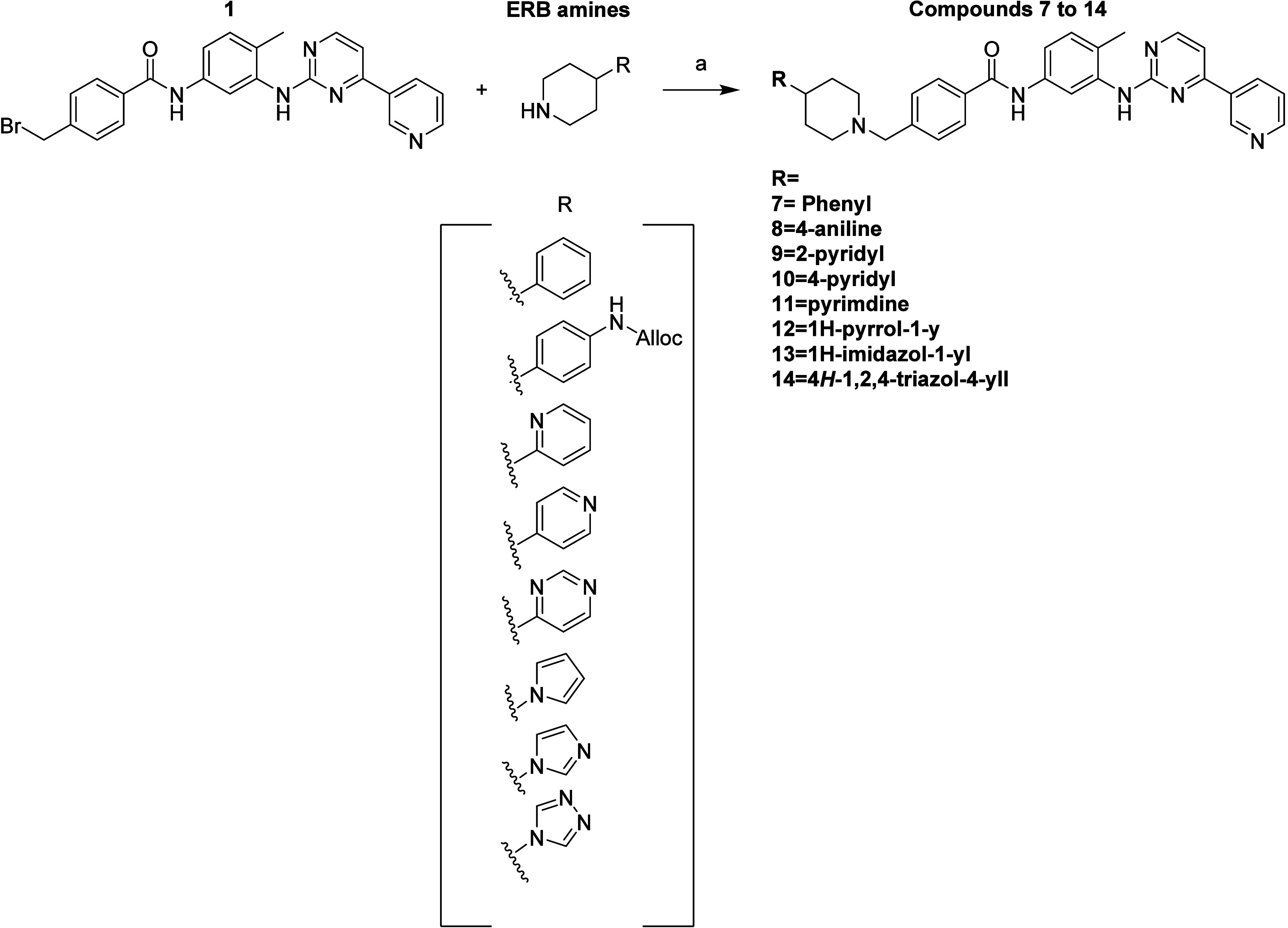
Synthesis of Imatinib Derivatives **7** to **14** ^a^

a Reagents and conditions: a) K_2_CO_3_, DMF, reflux, overnight.

### Cytotoxicity Screening in K562 Wild-Type Cells

Annexin V/7-AAD assays were used to assess cytotoxicity in K562 cells following 72-h exposure. Compounds **8**, **9**, **11**, and **13** induced apoptosis in a dose-dependent manner, while compounds **7**, **10**, **12**, and **14** were inactive. Compound **8** exhibited an LC_50_ of 2.29 μM, comparable to imatinib (2.64 μM), indicating that ERB modification preserved cytotoxic efficacy.^24^ Compound **9** (LC_50_ = 6.1 μM) also demonstrated activity despite modest kinase inhibition, suggesting potential differences in intracellular accumulation. Inactive analogs likely suffered from poor permeability or suboptimal physicochemical properties. These findings underscore the importance of ERB orientation and structure in influencing bioavailability. Notably, divergence between kinase inhibition and cytotoxicity suggested that efflux susceptibility and membrane permeability contribute substantially to whole-cell activity^7,25^ (Figure 3).

**Figure 3:**
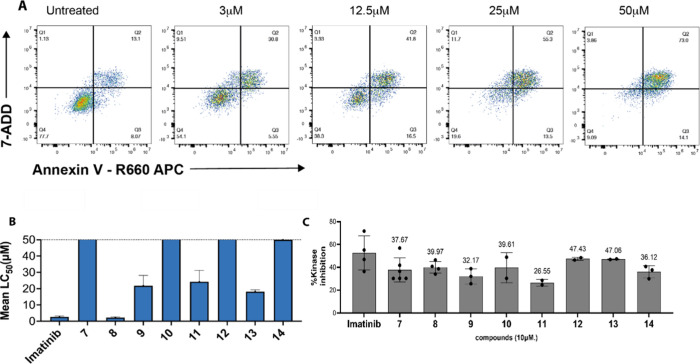
Imatinib derivatives induce dose response apoptosis in a leukemia cell line. (A) Annexin V and 7-AAD bivariate plots from K562 wild-type cells treated with increasing concentrations of compound **8**. (B) Comparative analysis shown by bar chart of imatinib and ERB-modified imatinib derivatives in K562 wild-type cell lines; compound **8** was the most potent in this cell line. Values represent mean LC_50_ value of *n* = 3 independent experiments with standard deviation. (C) *In vitro* BCR-ABL1 kinase inhibition by imatinib and ERB-modified analogs. Dose–response curves show the percentage inhibition of BCR-ABL1 kinase activity by imatinib and selected derivatives. Data represent mean ± SD from at least three independent experiments.

### Kinase Inhibition Assay

In the initial ABL1 kinase assay, imatinib inhibited approximately 53% of ABL1 activity at 10 μM, while ERB analogs showed variable inhibition ranging from 26% to 47% (Figure 3C). Compound **11** displayed the highest level of ABL1 inhibition but modest cytotoxicity, whereas compound **9** exhibited relatively weak inhibition yet remained cytotoxic, indicating that intracellular drug levels, rather than biochemical inhibition alone, are key determinants of cellular efficacy. Compound **8** retained cytotoxicity despite moderate inhibition, consistent with enhanced intracellular accumulation due to efflux resistance breaker modification. These findings emphasize the need to interpret kinase inhibition data in parallel with cellular assays, particularly in resistant cancer models where transporter expression influences pharmacodynamic outcomes.

In the extended kinase selectivity assays, imatinib, compound **8**, and compound **9** were evaluated against PDGFRα (D842Y) and CSF-1R. Both compounds **8** and **9** demonstrated inhibitory activity that was broadly comparable to imatinib across these kinases (Figures S7a and S7b). These results suggest that efflux resistance breaker modification did not compromise the ability of the analogs to engage clinically relevant kinase targets.

### Evaluation in Imatinib Resistant (K562/DOX) Cells

In K562/DOX cells, which overexpress P-gp,^4,5^ imatinib’s LC_50_ increased from 2.64 to 6.65 μM, consistent with efflux-mediated resistance (Figure 4). Most analogs showed reduced potency, but compound **8**’s LC_50_ rose only to 5.29 μM, indicating partial evasion of P-gp. Compound **9** demonstrated equipotency in resistant cells (LC_50_ = 20.3 μM vs 21.8 μM in K562 wild-type cells), and more strikingly compounds **10** and **12** showed increased potency in K562/DOX cells (LC_50_ values = 37.5 and 17.8 μM respectively vs >50 μM in K562 wild-type cells). These results support the concept of our ERB design as a valid strategy for mitigating P-gp efflux liability. Furthermore, compounds **9**, **10** and **12** may exemplify mechanistically distinct agents capable of exploiting other vulnerabilities in resistant phenotypes.

**Figure 4:**
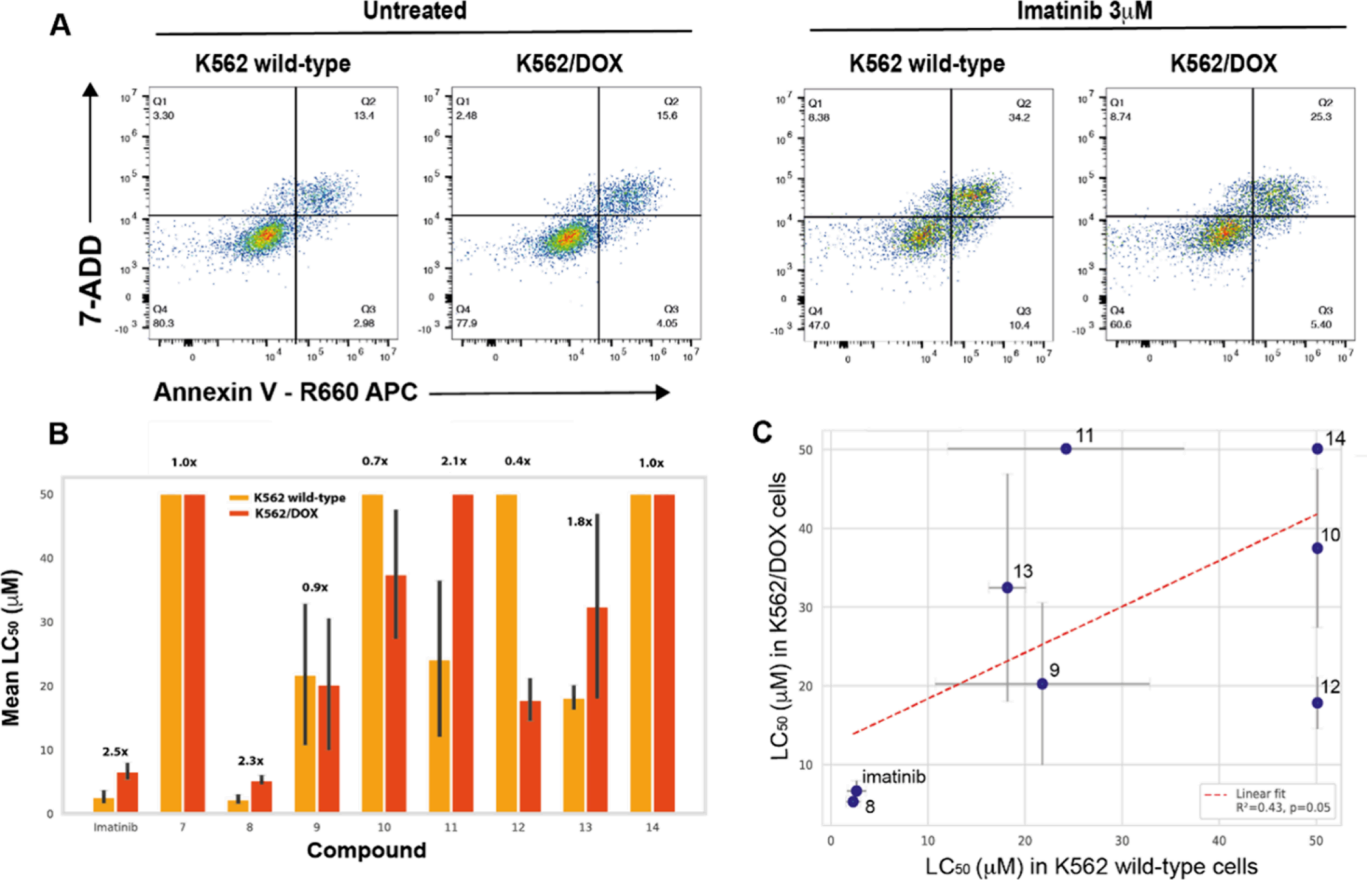
Resistance observed in K562/DOX cell lines for imatinib and ERB-modified derivatives. (A) Flow cytometric plots where the percentage of live cells is depicted in Q4 (Annexin V-/7-AAD-). Relative cell death was measured after incubation with increasing concentrations of imatinib for 72 h. (B) Comparative analysis of cytotoxicity of imatinib and derivatives in wild-type and resistant cell lines revealed significant differential sensitivity to some compounds. Each bar represents the mean LC_50_ value (*n* = 3 with two technical repeats ± SEM), with the relative resistance observed in K562/DOX cells expressed as fold-change in LC_50_ value for each compound. (C) Relationship between cytotoxicity observed in K562/DOX and K562 wild-type cell lines.

### P-gp Expression and Function

Flow cytometry confirmed >20-fold upregulation of P-gp in K562/DOX cells relative to parental cells.^4^ Functional activity was validated using rhodamine 123 efflux, which was reversed by verapamil cotreatment, confirming P-gp-mediated transport. This dual validation of expression and function affirms the relevance of K562/DOX as a resistance model. Compounds **8** and **9** maintained efficacy in P-gp–overexpressing cells, indicating reduced susceptibility to efflux (Figure 5). The use of this model enabled meaningful assessment of ERB effects on transporter susceptibility, further supporting these compounds’ therapeutic potential.

**Figure 5:**
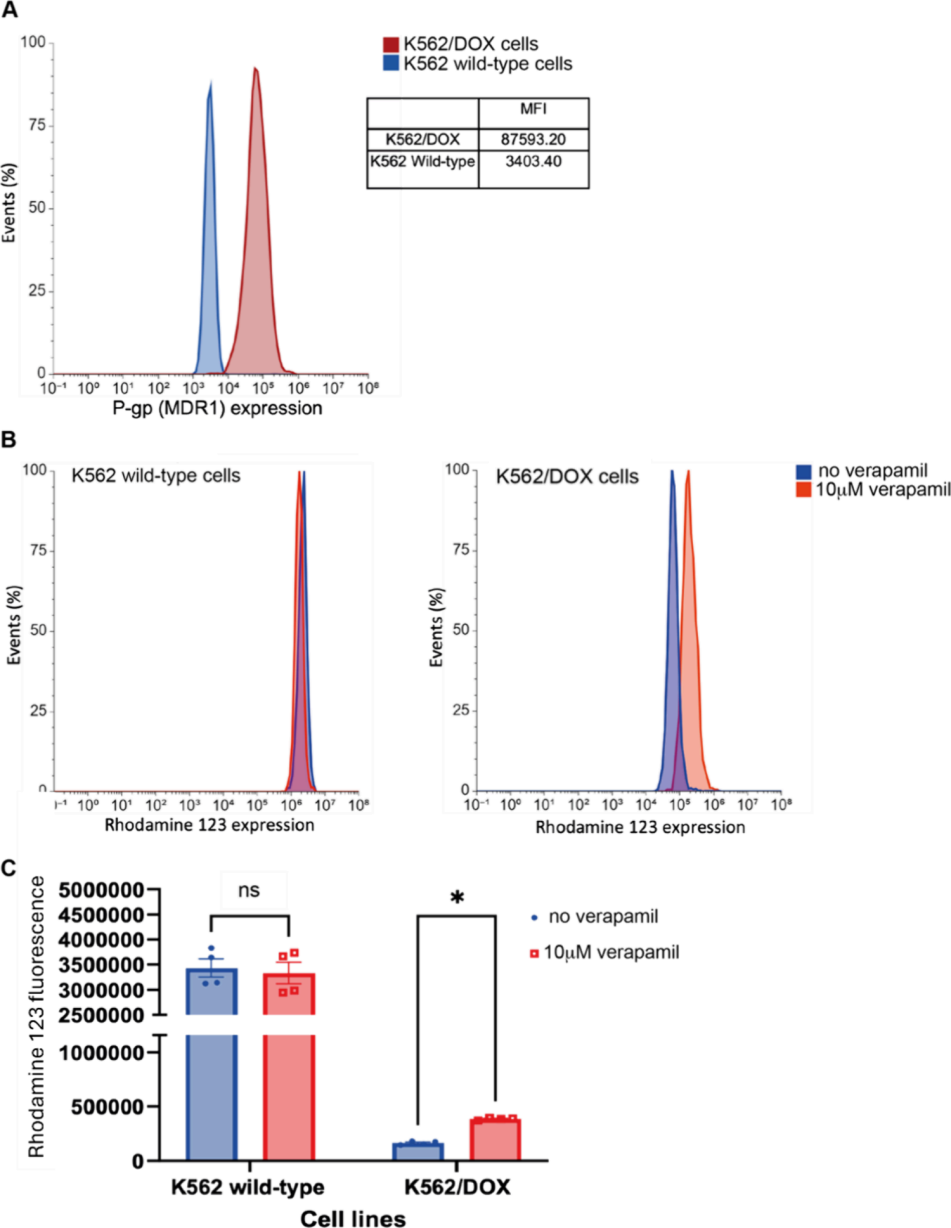
Cell surface expression and activity of P-gp in the different cell lines. (A) Cell surface expression of P-gp in K562 wild-type and K562/DOX cell lines. The mean fluorescence intensity of each histogram was used to quantify the relative P-gp expression of each cell line; K562/DOX cells show >25-fold increase in P-gp expression. (B) The overlaid histograms of intracellular rhodamine 123 (Rh123) expression illustrate the effects of verapamil blockade of P-gp on the intracellular Rh123 accumulation in K562 wild-type (Left) and K562/DOX cells (Right). (C) The data show that K562 wild-type cells accumulate significantly more Rh123 than K562/DOX cell, which was associated with a > 25-fold lower P-gp expression. Treatment with the P-gp blocker, verapamil (10 μM for 30 min) did not significantly alter Rh123 in K562 wild-type cells but caused a significant increase in intracellular Rh123 in K562/DOX cells (*p* < 0.01, *n* = 3, paired *t* test).

### Accumulation of ERB-Analogs in K562 Wild-Type and K562/DOX Cell Lines

To evaluate the impact of ERB modification on reducing the efflux liability of the imatinib scaffold, an LCMS-based accumulation assay was performed in both P-gp-overexpressing K562/DOX cells and wild-type K562 cells. Imatinib exhibited significantly reduced intracellular accumulation in the P-gp overexpressing K562/DOX cell line compared with the wild-type K562 cells (5.60 μg/mL versus 7.28 μg/mL) (Figure 6). This reduction corresponded to an efflux ratio of 1.30 and a 23.1% decrease in intracellular concentration, with the difference reaching statistical significance (Mann–Whitney test, *p* = 0.041). These findings are consistent with imatinib being a substrate of P-gp and subject to active efflux in resistant cells.

**Figure 6:**
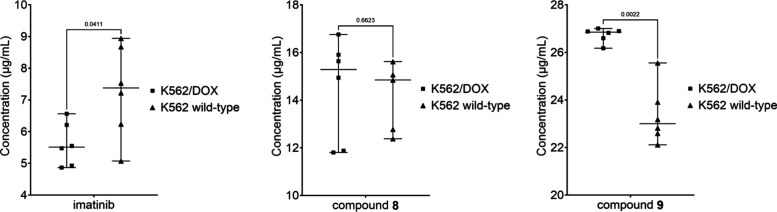
Intracellular accumulation of imatinib and ERB-modified analogs, compounds **8** and **9**, in K562 wild-type and P-gp–overexpressing K562/DOX cells.

In contrast, the efflux resistance breaker–modified analogs demonstrated markedly different behavior. Compound **8** showed no evidence of P-gp–mediated efflux, as intracellular concentrations in K562/DOX and K562 wild-type cells were comparable (14.49 μg/mL versus 14.14 μg/mL), corresponding to an efflux ratio of 0.98 and a nonsignificant 2.5% increase in the resistant cells (*p* = 0.662). This indicates that compound **8** was unaffected by P-gp overexpression and maintained stable intracellular levels across both cell lines.

Interestingly, compound **9** accumulated to significantly higher levels in the P-gp–overexpressing K562/DOX cells relative to the wild-type cells (26.73 μg/mL versus 23.36 μg/mL), giving an efflux ratio of 0.87 and representing a 14.4% increase in intracellular concentration. This observation suggests that structural modification of the imatinib scaffold not only mitigated P-gp–mediated efflux but may also have conferred an enhanced ability to accumulate in resistant cells.

When compared directly within the resistant K562/DOX cells, compounds **8** and **9** achieved approximately 2.6-fold and 4.8-fold higher intracellular concentrations, respectively, relative to imatinib (Figure S8). Taken together, these findings demonstrate that targeted modification of the imatinib scaffold using efflux resistance breaker technology effectively reduced P-gp liability and significantly enhanced intracellular drug accumulation in resistant cells, thereby providing a promising strategy to overcome transporter-mediated drug resistance.

### Influence of P-gp on Cytotoxicity

Cytotoxicity assays with and without verapamil confirmed imatinib’s susceptibility to P-gp efflux: LC_50_ in resistant cells returned to wild-type levels upon P-gp inhibition.^4,8^ In contrast, compounds **8** and **13** showed minimal changes, indicating their reduced reliance on P-gp for intracellular retention (Figure 7). These results, aligned with docking data, suggest ERBs can attenuate P-gp recognition. Embedding efflux resistance directly into the drug structure avoids the toxicity of systemic efflux inhibitors^8^ and enhances selectivity. Future studies could investigate whether these compounds evade other transporters such as BCRP or MRP1.

**Figure 7:**
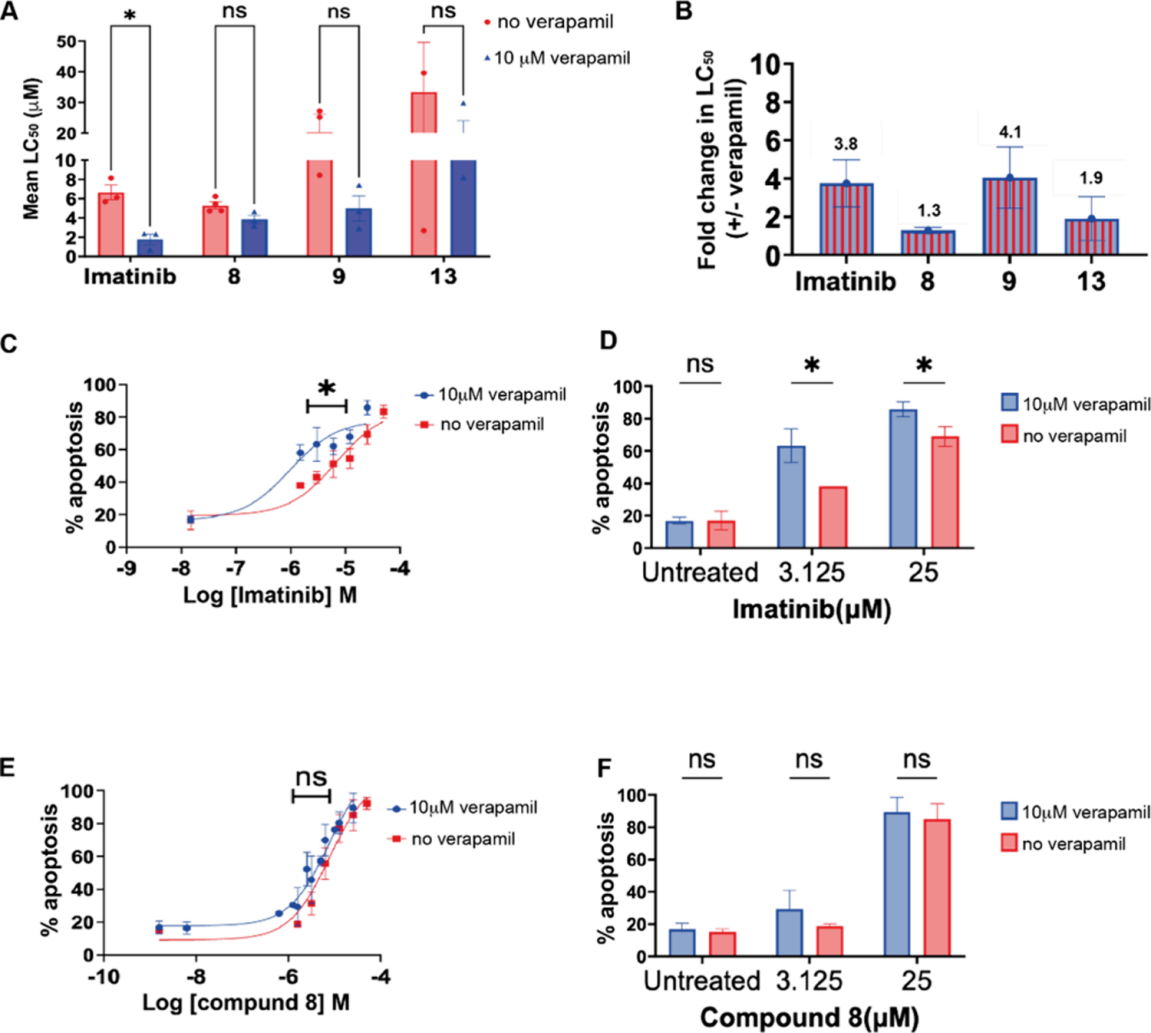
Impact of P-gp inhibition on the cytotoxic effect of imatinib and derivatives as determined by apoptosis assay in K562/DOX cells. (A) Summary data of the lead compounds in K562/DOX cell lines revealed significant differential sensitivity to imatinib following treatment with and without verapamil. In contrast, reversal of resistance was not significant for analog **8**, **9** and **13** after P-gp inhibition. (B) Efflux factor denoted by fold change in cytotoxicity with verapamil was lower for **13** and analog **8** compared to imatinib. Overlaid sigmoidal dose–response curves illustrating the comparative effects of (C) imatinib and (E) compound **8**(C) in K562/DOX cells after treatment with verapamil. The corresponding bar charts (D and F) show Annexin V/7AAD + cells for imatinib and **8** at two different concentrations. All *in vitro* experiments were performed in triplicate and data are presented as mean ± SEM.

### Toxicity in Nontumor Cells

Compound **8** was evaluated in nonmalignant B-lymphocytes and HaCaT keratinocytes. No significant toxicity was observed at concentrations up to 100 μM, yielding a toxicity index (TI) of 3.4 relative to K562 cells, which was similar to imatinib^18^ (Figure 8). These findings suggest that ERB incorporation does not confer off-target cytotoxicity. This contrasts with systemic P-gp inhibitors, which failed clinically due to toxicity.^8^ Compound **8**’s selectivity and safety profile make it a strong candidate for further preclinical evaluation.

**Figure 8:**
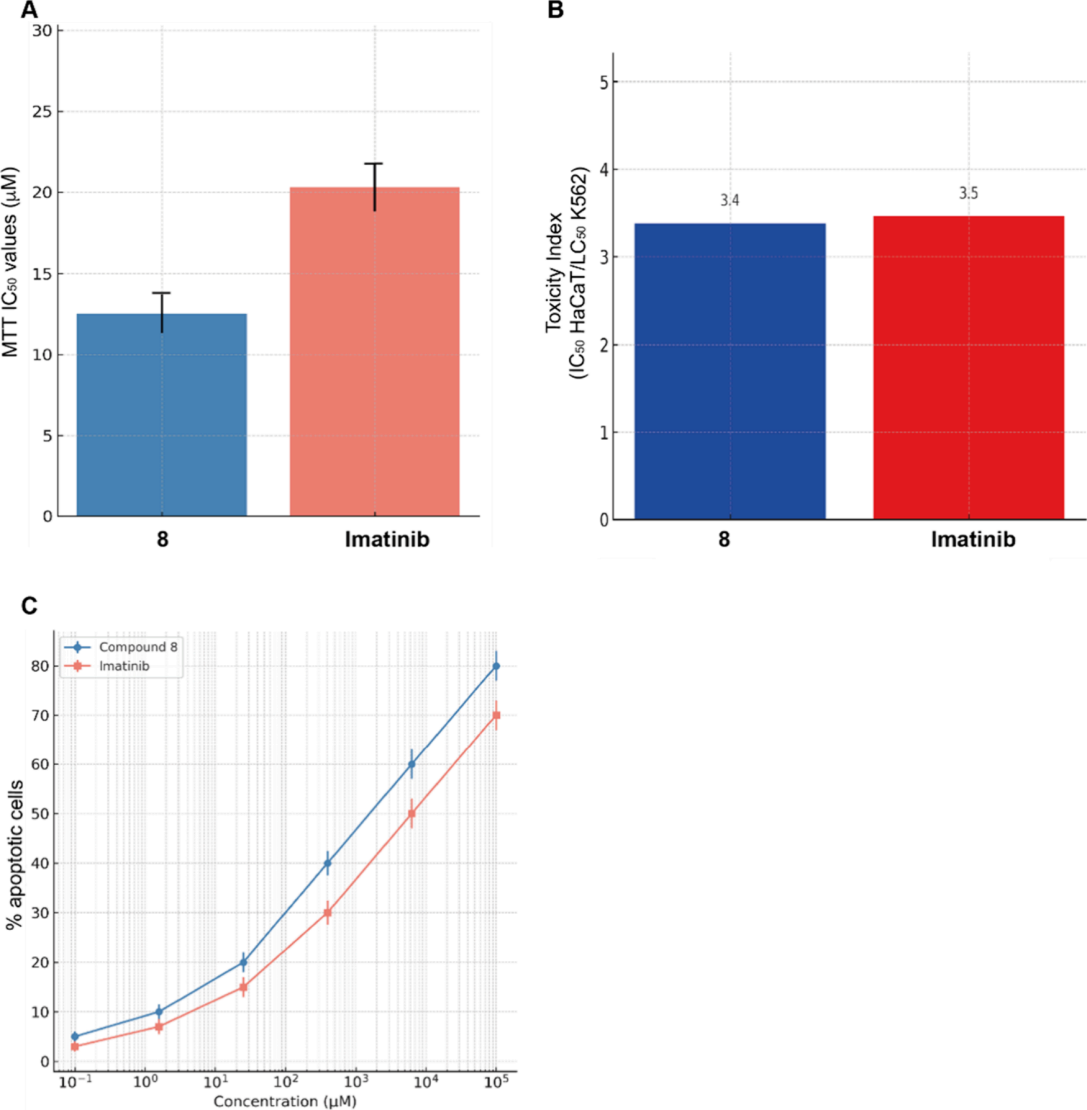
Comparative cytotoxicity and selectivity of compound **8** and imatinib. (A) *In vitro* cytotoxicity of compound **8** and imatinib in nontumorigenic human keratinocyte HaCaT cells, determined using MTT assay after 72 h of drug exposure across a concentration range of 0.1–100 μM. Bars represent the mean IC_50_ values ± standard deviation (SD) from three independent experiments. (B) Toxicity Index (TI) values for compound **8** and imatinib. TI is defined as the ratio of IC_50_ in HaCaT cells to LC_50_ in K562 wild-type leukemia cells. A higher TI indicates greater selective cytotoxicity toward cancer cells. (C) Apoptotic response of normal peripheral blood lymphocytes treated with compound **8** and imatinib for 72 h.

The development of chemotherapeutic agents that evade efflux transporters such as P-gp remains a significant challenge in overcoming multidrug resistance in cancer. Our strategy focused on incorporating ERB fragments into imatinib analogs to reduce P-gp-mediated efflux while preserving BCR-ABL1 inhibitory activity. The most promising compound from this study, compound **8**, maintained cytotoxic potency comparable to imatinib in wild-type K562 cells despite showing slightly reduced *in vitro* kinase inhibition. This apparent disconnect between biochemical and cellular activity highlights the importance of intracellular drug retention, particularly in the context of efflux-prone cell populations. Consistent with this observation, accumulation assays confirmed that compound **8** displayed improved intracellular retention in resistant cells compared with imatinib, supporting its reduced susceptibility to P-gp-mediated efflux. Compound **8**’s ability to partially evade efflux in K562/DOX cells, as shown by a modest increase in LC_50_ and limited reversal by verapamil, suggests that structural modifications at the piperazine terminus can influence P-gp interactions without compromising target engagement. The broader implication is that reduced kinase activity or on-target activity in general does not necessarily preclude therapeutic utility, particularly if intracellular exposure is improved. Sustained intracellular levels may not only enhance efficacy but also mitigate the emergence of secondary resistance mechanisms.

Another important consideration in the development of efflux-resistant agents is selectivity. Systemic P-gp inhibitors have historically failed due to off-target toxicity. In this study, compound **8** exhibited no significant cytotoxicity toward nonmalignant B-lymphocytes at concentrations up to 100 μM, supporting a favorable therapeutic index. This suggests that embedding efflux-evading features within the drug scaffold may provide a safer alternative to systemic transporter inhibition. These results offer proof of concept that ERB technology can be used to rationally design anticancer agents with reduced susceptibility to efflux. Although the ERB fragments applied here were originally derived from bacterial efflux inhibitor studies, they provided a useful starting point. The moderate potency of some analogs may reflect suboptimal fit within human transporter systems, underscoring the need for future iterations that tailor ERB structures to mammalian P-gp or BCRP binding pockets.

## Conclusion

This study demonstrates the feasibility and value of rational structural modification of imatinib to address transporter-mediated drug resistance in CML. By appending ERB moieties to the piperazine terminus—a known modifiable site without disrupting the core pharmacophore—we successfully generated a focused library of derivatives designed to evade P-gp recognition and efflux. The ERBs applied here are specific to the imatinib scaffold, but the overall ERB design concept encompasses a wider chemical space that can be tailored to other therapeutic scaffolds subject to efflux-mediated resistance.

Of the analogs synthesized and evaluated, compound **8** emerged as a lead candidate, exhibiting cytotoxic potency on par with imatinib in wild-type K562 cells while demonstrating a significantly reduced shift in LC_50_ in P-gp-overexpressing K562/DOX cells. Accumulation assays further confirmed improved intracellular retention of compound **8** in resistant cells relative to imatinib, consistent with its reduced susceptibility to efflux. Its minimal response to verapamil cotreatment and favorable *in silico* docking to the inhibitory access tunnel of P-gp further reinforce this reduced efflux liability. Additionally, compound **8** retained a strong therapeutic index in nontumor models, supporting its specificity and safety. Unexpectedly, compound **9** displayed enhanced cytotoxicity in the resistant cell line despite modest kinase inhibition, and accumulation assays revealed that it achieved significantly improved intracellular levels in both wild-type and resistant K562/DOX cells, which may explain its superior cytotoxic profile. This highlights a distinct opportunity to uncover unanticipated routes to overcoming resistance beyond classic target inhibition.

Taken together, these findings validate ERB incorporation as a rational design strategy to optimize tyrosine kinase inhibitors for activity in multidrug-resistant cancers. The dual benefit of maintained target specificity and improved intracellular retention presents a promising approach to overcoming one of the key clinical challenges in CML treatment. Future studies will focus on expanding this strategy to other scaffolds, refining ERB fragment design, and evaluating pharmacokinetics and efficacy in *in vivo* models.

## Experimental Section

### Synthesis

#### General Material and Methods

The synthetic building blocks, reagents, solvents, and chemicals required were obtained from Sigma-Aldrich, Fluorochem, Fisher Scientific and Alfa Aesar. Reaction mixtures were purified using traditional column chromatography utilizing standard glass column and silica gel as a stationary phase (Merck 60, 230–400 mesh ASTM). All reactions were performed under an inert atmosphere of nitrogen with oven-dried glassware unless otherwise mentioned. Precoated silica gel plates (Merck, silica gel 60 F254) were used to monitor the reaction progress by visualizing using UV light at 254 nM. Freeze-drying of intermediates/final compounds when required was performed, using a Lablyo Plus freeze-dryer. The melting point (m.p) values for final compounds in solid form were determined using a Stuart Melting Point Apparatus SMP30 (Stuart equipment, Cole-Parmer manufactures, UK). Final compounds were purified using High-performance liquid chromatography (HPLC) coupled to mass spectrometry. Waters Alliance 2695 separation system using water (solvent A) and acetonitrile (solvent B) as mobile phases using a Monolithic C18 50 × 4.60 mm column by Phenomenex was used for the analysis of the HPLC results. UV detection was performed by a diode array detector. Formic acid was maintained at concentrations of 0.1% to ensure acidic conditions throughout the analysis. Purity of all the compounds tested for their biological activity were confirmed (>95%) with two different HPLC analysis methods. Ten min (method A) and 5 min (method B) gradient was used to analyze the submitted samples. The flow rate for each method was set at 0.5 mL/min; 200 μL was split over a zero dead volume T piece which passed into the mass spectrometer at a wavelength range of the UV detector of the set at 220–500 nm. The function type used was a diode array (535 scans). The column type was a monolithic C18 50 × 4.60 mm column. Method A (5 min run): From 95% A/5% B to 10% A/90% B over three minutes. From 10% A/90% B to 5% A/95% B over 30 s. Held constant at 5% A/95% B for a further minute. From 5% A/95% B to 95% A/5% B over 30 s. Method B (10 min run): From 95% A/5% B to 50% A/50% B over three minutes. From 50% A/50% B to 20% A/80% B over two minutes. From 20% A/80% B to 5% A/95% B over 1.5 min. Held constant at 5% A/95% B for a further 1.5 min. From 5% A/95% B to 95% A/5% B over 0.2 min. Held constant at 95% A/5% B for a further 1.8 min. 1H and 13C nuclei nuclear magnetic resonance (NMR) analyses were obtained using a Spectro spin 400 MHz spectrometer (from Bruker) equipped with a Sample Xpress (from Bruker) autosampler system, using deuterated solvents for sample preparation. The obtained spectra were interpreted using Mnova software (Mnova 15, Mestrelab).

##### Synthesis of 4-(Bromo­methyl)-*N*-(4-methyl-3-((4-(pyridin-3-yl)­pyr­imidin-2-yl)­amino)​phenyl)​benzamide (1)

4-bromomethylbenzoic acid (1 equiv. 3.07g, 14.28 mMol) was added to a solution thionyl chloride (3 equiv. 5.09g, 42.83 mMol) and dimethylformamide (0.5 mL) at 0 °C. The solution was left to stir under reflux at 75 °C for 6 h after which excess of solvent distilled off under vacuum to yield 3g (12.85 mM, 90%) of 4-(bromomethyl)-benzoyl chloride, which was added to a stirred solution of 6-methyl-N1-(4-(20yridine-3-yl) pyrimidin-2-yl) benzene-1,3-diamine (1.10 equiv. 3.92g, 14.13 mMol) in 50 mL of dry chloroform and triethylamine (2.5 equiv. 4.5 mL, 32.12 mMol) at 0 °C. After 18 h, the solution was washed with cold deionized water and the aqueous layer was concentrated under reduced pressure and subsequently dried using an oven overnight at 55 °C, to yield 6g of intermediate 4-(bromomethyl)​-*N*-(4-methyl-3-​((4-(20yridine-3-yl) pyrimidin-2-yl) amino) phenyl) benzamide (1) (yield 98%).

4-(Bromomethyl)-*N*-(4-methyl-3-((4-(pyridin-3-yl)­pyrimidin-2-yl)­amino)­phenyl)­benzamide: (1) Yellow solid. ^1^H NMR (400 MHz, MeOD) δ 9.31 (dd, J = 2.2, 0.9 Hz, 1H), 8.65 (dd, J = 4.9, 1.6 Hz, 1H), 8.61 (ddd, J = 8.0, 2.3, 1.6 Hz, 1H), 8.49 (d, J = 5.2 Hz, 1H), 8.26 (d, J = 2.2 Hz, 1H), 8.10 (d, J = 8.4 Hz, 2H), 7.72 (d, J = 8.4 Hz, 2H), 7.57 (ddd, J = 8.1, 4.9, 0.9 Hz, 1H), 7.43 (dd, J = 8.2, 2.3 Hz, 1H), 7.39 (d, J = 5.2 Hz, 1H), 7.29 (d, J = 8.3 Hz, 1H), 4.60 (s, 2H), 2.34 (s, 3H). ^13^C NMR (101 MHz, DMSO) δ 165.1, 162.1, 161.6, 160.0, 151.9, 148.7, 138.3, 137.4, 134.9, 133.1, 132.7, 131.5, 128.7, 128.4, 124.3, 117.9, 117.4, 108.0, 52.8, 18.1. HPLC Method A: purity = 93% retention time 2.32 min, Method B: purity = 94% retention time 5.155 min. Mass ionization not observed.

##### General Procedure for Suzuki Coupling and Hydrogenation (5a-b)

The mixture of commercially available bromo substituted aromatic compounds 3a (4-bromoaniline) or 3b (2-bromopyridine) (1.0 equiv), tert-butyl 4-(4,4,5,5-tetra­methyl-1,3,2-dioxa­borolan-2-yl)-3,6-dihydro­pyridine-1­(2H)-carboxylate 2 (1.2 equiv), K_2_CO_3_ (3.0 equiv) and tetrakis Pd (PPh_3_)_4_ (0.02 to 0.1 equiv) was irradiated at 100 °C for 40 min under microwave conditions. The black residue formed was removed using Celite filtration and solvent was concentrated in vacuo followed by hydrogenation over Pd/C (10%, w/w) under pressure (40 psi) in a Parr hydrogenator apparatus overnight. After filtration using Celite, the solution was concentrated in vacuo to give 5a-b (>80% yield) which were carried forward to the next step without further purification.

##### Synthesis of Allyl­(4-(piperidin-4-yl)­phenyl)­carbamate (6c)

5a (1 equiv.,707 mg, 2.56 mMol) was dissolved in 10 mL of dry DCM and dry pyridine (0.8 equiv., 166 μL, 2.05 mM) and allyl chloroformate (0.95 equiv., 260 μL, 2.43 mM), was added at −10 °C and left to stir. After 2 h, once confirmed complete by TLC and LC-MS, the reaction mixture washed with copper sulfate solution (50 mL) to remove the pyridine. Once separated properly after half an hour, the organic layer was washed further with saturated aqueous NaHCO_3_ (100 mL) and brine (70 mL x 2). The organic phase was dried over MgSO_4_ and evaporated under vacuum using a rotary evaporator and the crude was purified using column chromatography mobile phase (mobile phase DCM 100% to 100% EA) affording pure 600 mg of sticky brown solid Alloc-protected 6a. 6a was dissolved in methanol and 4 N HCl in dioxane was added and the reaction left to stir for 1 h. The reaction mixture was concentrated using a rotary evaporator to give the 500 mg of alloc protected amine **6c** (Yield 89%, 2 steps).

Allyl­(4-(piperidin-4-yl) phenyl)­carbamate (**6c**): Light pink oil. ^1^H NMR (400 MHz,DMSO-d6) δ 7.41 (d, *J* = 8.2 Hz, 2H), 7.14 (d, *J* = 8.4 Hz, 2H), 6.02 – 5.92 (m, 1H), 5.41 – 5.21 (m, 2H), 4.59 (dt, *J* = 5.5, 1.4 Hz, 2H), 3.32 (d, *J* = 12.6 Hz, 2H), 3.08 – 2.73 (m, 3H), 1.59 (dtd, *J* = 14.5, 10.6, 3.9 Hz, 1H). ^13^C NMR (101 MHz, DMSO-d6) δ 153.4, 138.9, 137.7, 133.5, 127.0, 117.7, 64.8, 39.1, 38.9, 29.6, 24.8, 23.6. HPLC Method A: retention time 2.226 min. *m*/*z* (+ESI) *m*/*z* (+ESI) calc. for C_15_H_20_N_2_O_2_ (M)+ 260.15, found 261.1 ([M] + H)^+^.

##### Synthesis of 2-(Piperidin-4-yl)­pyridine (6b)

5b (1 equiv., 400 mg, 1.52 mMol) was dissolved in methanol and 4 N HCl in dioxane was added and the reaction left to stir for 1 h. The reaction mixture was concentrated using a rotary evaporator to give 240 mg (97% yield) of **6b**.

2-(Piperidin-4-yl)­pyridine (6b): Light yellow solid. ^1^H NMR (400 MHz, MeOD) δ 8.78 (d, J = 4.8 Hz, 1H), 8.61 (d, J = 6.9 Hz, 1H), 8.12 – 7.92 (m, 2H), 3.59 (d, J = 21.7 Hz, 1H), 3.17 (q, J = 7.2 Hz, 2H), 2.29 (d, J = 12.6 Hz, 2H), 2.05 – 1.81 (m, 2H), 1.16 (s, 2H). ^13^C NMR (101 MHz, MeOD) δ 147.7, 141.7, 125.8, 125.6, 43.4, 38.0, 27.4. HPLC Method A: retention time 0.746 min. *m*/*z* (+ESI) *m*/*z* (+ESI) calc. for C_10_H_14_N_2_ (M)^+^ 162.12, found 163.1 ([M] + H)^+^.

##### General Synthesis of Target Compounds (7–14)

To a stirred solution of the relevant 4-position substituted piperidine based amine (1 equiv), in 1–3 mL of DMF, was added K_2_CO_3_ (4 equiv) and left to stir for 1 h under reflux at 152–160 °C. To this heated solution under reflux, slowly, over 30 min, the benzamide core 1 (1 equiv) was added. The solution was left to stir under reflux for 6–12 h depending on the TLC monitoring. Once the starting materials were consumed, reaction was stopped, and quenched with 100 mL of cold water. The reaction mixture was then was washed with ethyl acetate (20 mL X 5). The organic layer was then further washed with Na_2_CO_3_ and then finally with NaCl and dried over MgSO_4_. The crude was then concentrated using rotary evaporator under reduced pressure and then purified either by HPLC or column chromatography to give the final imatinib analogs **7–14**.

##### 4-Methyl-3-((4-(pyridin-3-yl)­pyrimidin-2-yl)­amino)­phenyl-4-((4-phenylpiperidin-1-yl)­methyl)­benzamide (7)

The reaction afforded 55 mg (9%) o as brown solid (mp. 104 °C). R_f_ value (EA/TEA 1:99): 0.25; ^1^H NMR (400 MHz,DMSO-d6) δ 10.17 (s, 1H), 9.31 – 9.25 (m, 1H), 8.98 (s, 1H), 8.69 (dd, *J* = 4.8, 1.6 Hz, 1H), 8.54 – 8.45 (m), 8.09 (d, *J* = 2.2 Hz, 1H), 7.96 – 7.89 (m, 2H), 7.57 – 7.38 (m, 5H), 7.33 – 7.13 (m, 6H), 3.58 (s, 2H), 2.93 (d, *J* = 10.9 Hz, 2H), 2.48 (d, *J* = 7.7 Hz, 1H, 6), 2.23 (s, 3H), 2.14 – 2.04 (m, 2H), 1.79 – 1.61 (m, 4H). ^13^C NMR (101 MHz, DMSO-d6) δ159.9, 151.9, 148.7, 134.9, 130.5, 129.1, 128.8, 128.0, 127.2, 126.5, 124.3, 117.7, 117.2, 108.0, 54.2, 42.3, 33.6, 18.1. HRMS (EI, *m*/*z*): calc for C_35_H_34_N_6_O (M), 555.2867; found, 555.2850, Error: −2.12 ppm. HPLC Method A: retention time = 2.668 min. Method B: retention time = 6.092 min.

##### 4-((4-(4-Aminophenyl)­piperidin-1-yl)­methyl)-*N*-(4-methyl-3-((4-(23yridine-3-yl)​pyrimidin-2-yl)​amino)​phenyl)­benzamide (8)

The reaction afforded 25 mg (10%) as cream white powder (mp. 76.9 °C). ^1^H NMR (400 MHz, MeOD) δ 9.17 (d, J = 2.2 Hz, 1H), 8.52 (dd, J = 4.9, 1.6 Hz, 1H), 8.36 (d, J = 5.2 Hz, 1H), 8.11 (d, J = 2.2 Hz, 1H), 7.86 – 7.80 (m, 2H), 7.52 – 7.34 (m, 3H), 7.30 (dd, J = 8.3, 2.2 Hz, 1H), 7.24 (d, J = 5.3 Hz, 1H), 7.15 (d, J = 8.3 Hz, 1H), 6.93 – 6.80 (m, 2H), 6.57 (d, J = 8.3 Hz, 2H), 3.54 (s, 2H), 3.08 (q, J = 7.3 Hz, 1H), 2.98 – 2.87 (m, 2H), 2.21 (s, 3H), 2.06 (td, J = 10.6, 3.6 Hz, 2H), 1.81 (s, 1H), 1.69 – 1.61 (m, 3H). ^13^C NMR (101 MHz, MeOD) δ 158.9, 150.4, 147.6, 137.6, 136.8, 136.0, 135.4, 134.0, 130.2, 129.5, 127.9, 127.2, 126.9, 124.0, 117.3, 117.1, 115.6, 107.3, 62.5, 54.0, 41.4, 29.3, 16.4. HRMS (EI, *m*/*z*): calc for C_35_H_35_N_7_O (M), 570. 29758; found, 570. 29758, Error: −0.29 ppm. HPLC Method A: retention time = 2.227 min. Method B: retention time = 4.792 min.

##### 4-Methyl-3-((4-(pyridine-3-yl)­pyrimidin-2-yl)­amino)­phenyl-4-((4-(pyridine-2-yl)­piperidin-1-yl)­methyl)­benzamide (9)

The reaction afforded 55 mg (9%) as a light-yellow oil. R_f_ value (EA/TEA 1:99): 0.4. ^1^H NMR (400 MHz,DMSO-d6) δ 10.18 (s, 1H), 9.28 (s, 1H), 8.97 (s, 1H), 8.69 (d, *J* = 4.8 Hz, 1H), 8.54 – 8.44 (m, 3H), 8.15 (s, 1H), 7.98 – 7.91 (m), 7.70 (td, *J* = 7.7, 2.0 Hz, 1H), 7.56 – 7.46 (m, 4H), 7.43 (dd, *J* = 5.6, 1.9 Hz, 1H), 7.28 (d, *J* = 7.8 Hz, 1H), 7.21 (dd, *J* = 7.8, 2.7 Hz, 2H), 3.67 (s, 2H), 2.97 (s, 1H), 2.67 (ddt, *J* = 20.9, 15.4, 6.5 Hz, 1H), 2.55 (t, *J* = 1.2 Hz, 1H), 2.30 (s, 1H), 2.23 (m, 3H), 1.89 – 1.72 (m, 4H). ^13^C NMR (101 MHz, DMSO) δ 165.7, 164.8, 163.6, 162.1, 161.7, 159.9, 151.8, 149.3, 148.7, 141.9, 138.3, 137.7, 137.0, 134.9, 134.4, 132.7, 130.5, 129.6, 129.3, 128.2, 128.1, 124.3, 122.0, 121.7, 117.7, 117.2, 108.0, 62.1, 53.7, 43.7, 31.7, 18.1. HRMS (EI, *m*/*z*): calc for C_34_H_34_N_7_O (M), 556.28194; found, 556. 2800, Error: −3.54 ppm. HPLC Method A: retention time = 2.202 min. Method B: retention time = 4.784 min.

##### 4-Methyl-3-((4-(pyridin-3-yl)­pyrimidin-2-yl)­amino)­phenyl-4-((4-(pyridin-4-yl)­piperidin-1-yl)­methyl)­benzamide (10)

The reaction afforded 30 mg (12%) as a cream solid powder (mp. 69 °C). ^1^H NMR (400 MHz,DMSO-d6) δ 10.19 (s, 1H), 9.28 (d, *J* = 2.3 Hz, 1H), 8.98 (s, 1H), 8.69 (dd, *J* = 4.8, 1.6 Hz, 1H), 8.51 (d, *J* = 5.1 Hz, 1H), 8.52 – 8.43 (m, 3H), 8.15 (s, 1H), 8.09 (d, *J* = 2.2 Hz, 1H), 7.93 (d, *J* = 7.9 Hz, 2H), 7.56 – 7.46 (m, 4H), 7.43 (d, *J* = 5.2 Hz, 1H), 7.28 (d, *J* = 5.8 Hz, 2H), 7.21 (d, *J* = 8.3 Hz, 1H), 3.64 (s, 2H), 2.88 (m, 3H), 2.23 (s, 3H), 2.20 – 2.10 (m, 2H), 1.82 – 1.62 (m, 4H). 13C NMR (101 MHz, DMSO) δ 165.7, 163.6, 162.1, 161.7, 159.9, 155.1, 151.9, 150.1, 148.7, 142.1, 138.3, 137.7, 134.9, 134.4, 132.7, 130.5, 129.3, 128.1, 124.3, 122.8, 117.7, 117.2, 108.0, 62.1, 53.6, 41.2, 40.9, 32.3, 18.1. HRMS (EI, *m*/*z*): calc for C_34_H_33_N_7_O (M), 556.2819; found, 556.2802, Error: --3.12 ppm. HPLC Method A retention time = 2.107 min. Method B: retention time = 4.165 min.

##### 4-Methyl-3-((4-(pyridin-3-yl)­pyrimidin-2-yl)­amino)­phenyl-4-((4-(pyrimidin-2-yl)­piperidin-1-yl)­methyl)­benzamide (11)

The reaction afforded 30 mg (12%) as a light brow solid powder (mp. 115.5 °C). R_f_ value (EA/MeOH 80:20): 0.5^1^H NMR (400 MHz,DMSO-d6) δ 10.21 (s, 1H), 9.28 (d, *J* = 2.3 Hz, 1H), 8.98 (s, 1H), 8.74 (d, *J* = 4.9 Hz, 2H), 8.69 (dd, *J* = 4.8, 1.6 Hz, 1H), 8.54 – 8.45 (m, 2H), 8.09 (d, *J* = 2.2 Hz, 1H), 7.94 (d, *J* = 8.0 Hz, 2H), 7.56 – 7.40 (m, 4H), 7.34 (t, *J* = 4.9 Hz, 1H), 7.21 (d, *J* = 8.3 Hz, 1H), 3.60 (s, 2H), 2.92 (d, *J* = 11.0 Hz, 2H), 2.81 (s, 1H), 2.23 (s, 3H), 2.13 (s, 2H), 1.91 (s, 2H), 1.35 (d, *J* = 6.3 Hz, 1H), 1.23 (d, *J* = 3.2 Hz, 4H). ^13^C NMR (101 MHz, DMSO-d6) δ 159.9, 157.6, 151.8, 148.6, 134.9, 130.4, 129.08, 128.1, 124.2, 119.7, 117.7, 117.2, 107.9, 62.4, 53.5, 44.9, 32.0,18.1. HRMS (EI, *m*/*z*): calc for C_33_H_32_N_8_O (M), 557.2772; found, 557.2758, Error: −3.12 ppm. HPLC Method A retention time = 2.347 min. Method B: retention time = 5.015 min.

##### 4-((4-(1*H*-Pyrrol-1-yl)­piperidin-1-yl)­methyl)-*N*-(4-methyl-3-((4-(pyridin-3-yl)­pyrimidin-2-yl)­amino)­phenyl)­benzamide (12)

The reaction afforded 30 mg (46%) as a light brow solid powder (mp. 112.5 °C). R_f_ value (DCM/MeOH 95:5): 0.6. ^1^H NMR (400 MHz,DMSO-d6) δ 10.18 (s, 1H), 9.28 (d, *J* = 2.3 Hz, 1H), 8.98 (s, 1H, 11), 8.69 (dd, *J* = 4.8, 1.7 Hz, 1H), 8.54 – 8.44 (m, 2H), 8.09 (d, *J* = 2.2 Hz, 1H), 7.93 (d, *J* = 7.9 Hz, 2H), 7.56 – 7.40 (m, 5H), 7.21 (d, *J* = 8.3 Hz, 1H), 6.83 (t, *J* = 2.1 Hz, 2H), 6.03 – 5.94 (m, 2H), 3.89 (tt, *J* = 10.3, 5.0 Hz, 1H), 3.58 (s, 2H), 2.91 (dd, *J* = 11.9, 3.5 Hz, 2H), 2.23 (s, 3H), 2.11 (td, *J* = 11.5, 3.2 Hz, 2H), 1.95 – 1.78 (m, 4H). ^13^C NMR (101 MHz, DMSO) δ 165.7, 162.1, 161.7, 159.9, 151.9, 148.7, 146.7, 142.7, 138.3, 137.7, 134.9, 134.3, 132.7, 130.5, 129.1, 128.1, 128.0, 126.5, 124.3, 119.1, 119.0, 119.0, 117.7, 117.2, 108.0, 107.8, 107.8, 107.7, 62.0, 56.4, 52.8, 43.3, 33.8, 30.9, 29.5, 18.1. HRMS (EI, *m*/*z*): calc for C_33_H_33_N_7_O (M), 544.2819; found, 544.2803, Error: −3.00 ppm. HPLC Method A: retention time = 2.572 min. Method B: retention time = 5.622 min.

##### 4-((4-(1*H*-Imidazol-1-yl)­piperidin-1-yl)­methyl)-*N*-(4-methyl-3-((4-(pyridin-3-yl)­pyrimidin-2-yl)­amino)­phenyl)­benzamide (13)

The reaction afforded 90 mg (30%) as a light brown sticky oil. R_f_ value (EA/MeOH 80:20): 0.2. ^1^H NMR (400 MHz,DMSO-d6) δ 10.09 (s, 1H), 9.19 (dd, *J* = 2.3, 0.8 Hz, 1H), 8.89 (s, 1H), 8.60 (dd, *J* = 4.8, 1.6 Hz, 1H), 8.46 – 8.36 (m, 2H), 8.00 (d, *J* = 2.2 Hz, 1H), 7.95 (s, 1H), 7.87 – 7.81 (m, 2H), 7.64 (q, *J* = 1.2 Hz, 1H), 7.47 – 7.26 (m, 5H), 7.19 (q, *J* = 1.3 Hz, 1H), 7.12 (d, *J* = 8.4 Hz, 1H), 6.80 (dt, *J* = 2.2, 1.1 Hz, 1H), 4.32 – 4.16 (m, 1H), 3.51 (s, 2H), 3.12 – 3.01 (m, 2H), 3.01 – 2.89 (m, 1H), 2.63 (dd, *J* = 12.9, 3.1 Hz, 1H), 2.14 (s, 3H), 2.09 – 1.99 (m, 2H), 2.03 – 1.90 (m, 1H), 1.64 (dtd, *J* = 36.9, 12.3, 7.5 Hz, 1H’). ^13^C NMR (101 MHz, DMSO) δ 165.7, 161.7, 159.9, 151.9, 148.7, 142.6, 138.3, 134.9, 134.3, 132.7, 130.5, 129.1, 128.7, 128.1, 124.3, 118.0, 117.2, 108.0, 61.8, 54.5, 52.5, 29.5, 18.1. HRMS (EI, *m*/*z*): calc for C_32_H_32_N_8_O (M^+^), 545.2772; found, 545.2764, Error: −1.39 ppm. HPLC Method A: retention time = 4.325 min. Method B: retention time = 2.097 min.

##### 4-((4-(4*H*-1,2,4-Triazol-4-yl)­piperidin-1-yl)­methyl)-*N*-(4-methyl-3-((4-(pyridin-3-yl)­pyrimidin-2-yl)­amino)­phenyl)­benzamide (14)

The reaction afforded 20 mg (30%) as a light-yellow solid powder. R_f_ value (DCM/MeOH 95:5): 0.1. ^1^H NMR (400 MHz, MeOD) δ 9.30 (d, *J* = 2.2 Hz, 1H), 8.68 – 8.57 (m, 4H), 8.49 (d, *J* = 5.3 Hz, 1H), 8.22 (d, *J* = 2.2 Hz, 1H), 7.95 (d, *J* = 8.0 Hz, 2H), 7.61 – 7.51 (m, 3H), 7.46 – 7.35 (m, 2H), 7.28 (d, *J* = 8.2 Hz, 1H), 4.29 (td, *J* = 11.6, 5.9 Hz, 1H), 3.72 (s, 2H), 3.08 (d, *J* = 11.9 Hz, 2H), 2.36 – 2.26 (m, 5H), 2.17 (d, *J* = 11.9 Hz, 2H), 2.08 (tt, *J* = 13.0, 6.6 Hz, 2H). ^13^C NMR (101 MHz, MeOD) δ 158.9, 150.4, 147.7, 141.6, 137.6, 135.4, 134.1, 130.2, 129.1, 128.0, 127.4, 124.0, 117.4, 117.1, 107.3, 61.6, 53.9, 51.8, 32.3, 16.4. HRMS (EI, *m*/*z*): calc for C_31_H_31_N_9_O (M^+^), 546.2724; found, 546.2706, Error: −3.36 ppm. HPLC Method A: retention time = 2.151 min. Method B: retention time = 4.705 min.

#### Biological Experiments

K562 wild-type, K562/DOX, OCI-AML3, HaCaT and healthy B cell cytotoxicity screening. Healthy B-lymphocytes were obtained from healthy donor blood samples using density gradient centrifugation with Histopaque. Informed consent was obtained from all human participants in this study and experiments were performed in accordance with ethical approval granted by the local research ethics committee (17/SW/0263). The primary cells were used within 24 h of sample preparation and were maintained in Roswell Park Memorial Institute (RPMI) media (10% fetal bovine serum (FBS), 1% glutamate and 1% penicillin/streptomycin (P/S). All human cell lines were purchased from American Type Culture Collection (ATCC, USA), except K562/DOX cell line which was gifted by Professor Gambacorti-Passerini (University of Milan Bicocca, Italy). K562 wild-type and K562/DOX cells were maintained in RPMI 1640 media with 10% FBS, 1% P/S and 1% l-glutamine (complete media) between 0.5 × 10^6^ and 1 × 10^6^ cells/mL. HaCaT cells were maintained in Dulbecco’s modified Eagle’s media (DMEM) with high glucose containing 10% FBS, 1% P/S and 1% l-Glutamine (complete media) at concentrations between 0.5 × 10^6^ and 2 × 10^6^ cells/mL.

All cells were maintained at 37 °C in atmospheric conditions of 5% CO_2_. Cytotoxicity screening was carried out in triplicate with these cells at a cell density of 0.5 × 10^6^ cells/mL. Cells were treated with test compounds at a range of concentrations to establish LC_50_ values. Cells were incubated with the compounds for 72 h before being harvested by centrifugation. Subsequently, cells were labeled with Annexin V and 7-AAD (bioscience) in accordance with the manufacturer’s instructions. Following staining with FITC-labeled annexin V and 7-AAD, samples were analyzed using CytExpert software on a CytoFLEX LX flow cytometer (Beckman Coulter) to determine relative levels of cell death in the sample. Prism 10 software was then used to analyze the data to obtain dose–response curves.

#### MTT Assay

HaCaT cells were seeded in a 96-well plate in DMEM at a density of 3 × 10^3^ cells/well and incubated for 24 h at 37 °C in a humidified incubator with 5% CO_2_. The cells were then treated with a series of drug concentrations (0.1–100 μM) and incubated for a further 72 h at 37 °C. After treatment, the media in each well, including control wells, was replaced with MTT reagent (0.1 mg/well in media) and incubated for 4 h at 37 °C. Following incubation, the MTT reagent was carefully removed, and 200 μL of DMSO was added to each well to solubilize the formazan crystals. The plate was shaken for 10 min, and the optical density (OD) was measured at 570 nm using a microplate reader.

#### P-gp Expression and Functional Assay

##### Preliminary P-gp Screening

The cell lines were screened for cell surface expression of P-glycoprotein (MDR-1). Analysis of surface level P-gp expression was conducted using a CytoFLEX LX flow cytometer. Cells were harvested and aliquots of 1 × 10^6^ cells were placed into FACS tubes. For each cell line, 2 aliquots of cells, one for unstained control and the other for P-gp staining were prepared. The cells were resuspended in FACS staining buffer (100 μL/tube) and 5 μL/10^6^ cells of MDR1 Brilliant violet 421-labeled antibody was added to each of the tubes followed by brief vortexing. The cells were incubated for 15 min at room temperature in the dark before washing the cells in 1 mL FACS staining buffer 300x*g* for 5 min. The cells were then resuspended in 100 μL of flow cytometry staining buffer and analyzed by flow cytometry (CytoFLEX LX, Beckman Coulter). 10,000 events were acquired for each sample.

##### Rhodamine 123 Accumulation Assay to Determine P-gp Activity

P-gp activity was assessed by measuring the intracellular accumulation of rhodamine 123 (Rh123). The cells were incubated at 37 °C, in 24-well plates at a concentration of 1 × 10^6^ cells/mL, with 1 μM Rh123 for 30 min under two experimental conditions with and without the addition of the P-gp inhibitor, verapamil (10 μM). The cells were then centrifuged for 300x*g* for 5 min to remove media with Rh123 and resuspended in cold 100 μL PBS. Quantification of Rh123 fluorescence was determined by flow cytometry using a CytoFLEX LX instrument (Beckman Coulter). 10,000 events were acquired for each sample.

##### Effect of P-gp Activity on Apoptosis Using Verapamil

Cells were seeded onto 96-well plates at a concentration of 0.5 × 10^6^ cells/well in a final volume of 200 μL. After 30 min of incubation with the P-gp inhibitor, verapamil, at a concentration of 10 μM, compounds were added at different concentrations ranging from 1.6 μM to 50 μM for imatinib derivatives with a 2-fold serial dilution across the plate. After a 72-h incubation period with the test compounds, both with and without the inhibitor, apoptotic cells were evaluated using the Annexin V assay to determine the extent of apoptosis.

#### Accumulation Assay

K562 wild-type and K562/DOX cells were seeded in 6-well plates at a density of 1 × 10^6^ cells per well in a final volume of 2 mL and cultured for 24 h at 37 °C in a humidified incubator with 5% CO_2_. The cells were then treated with 50 μM of the drug and incubated for 2 h. After incubation, the cells were harvested, washed with ice-cold phosphate-buffered saline (PBS), and pelleted. Intracellular drug was extracted by lysing the cells with 400 μL of methanol, aided by sonication. The lysates were centrifuged at 7200 rcf, and the supernatant was collected for analysis by LC-MS/MS. Drug quantification was performed using an external calibration method. Stock solutions of each compound were prepared in methanol and used to generate calibration standards in methanol/DMSO (70:30, %v/v). Experimental samples were diluted in the same solvent mixture prior to analysis.

Chromatographic separation was performed on a Shimadzu Nexera XR ultrahigh-performance liquid chromatography (UHPLC) system comprising two LC-20AD pumps, a DGC degasser, a SIL-30AC autosampler, a CBM-20A controller, and a column oven. The system was fitted with an Ascentis Express C18 column (5 cm × 2.1 mm, 2.7 μm particle size). Elution was performed with a binary solvent system: eluent A, 0.1% formic acid in water; eluent B, 0.1% formic acid in methanol. The gradient program was as follows: 5% B for 0.5 min, increased to 40% B over 3.5 min, increased to 95% B over 2 min, held at 95% B for 1.5 min, then returned to 5% B at 8 min and held for 3 min for re-equilibration. The mobile phase flow rate was 0.21 mL/min. The column oven was maintained at 40 °C and the autosampler at 10 °C.

The UHPLC system was coupled to a Shimadzu 8060 triple quadrupole mass spectrometer with an electrospray ionization (ESI) source operating in positive ion mode. Instrument settings were: nebulizing gas flow, 3.0 L/min; drying and heating gas flow, 10 L/min each; interface voltage, 4.5 kV; interface temperature, 300 °C; desolvation temperature, 526 °C; desolvation line temperature, 250 °C; and heat block temperature, 400 °C. Multiple reaction monitoring (MRM) transitions (Table S2) were used for quantification with a dwell time of 100 ms per transition. Data were normalized against standards prepared using the initial drug concentration in the lysis solution. Statistical significance of differences in intracellular accumulation between drugs and cell lines was assessed using the nonparametric Mann–Whitney test (n = 6).

#### Kinase Assay

Kinase inhibition was assessed using luminescent ADP-Glo–based assays. For ABL1, reactions contained recombinant ABL1 kinase (2.5 ng/reaction), substrate peptide (1 μg), ATP (25 μM), and test inhibitors (1.5–50 μM) in commercial kinase buffer, giving a total volume of 25 μL. Imatinib was included as a positive control. No-enzyme and no-compound reactions served as 0% and 100% activity controls, respectively. Luminescence was quantified using a Tecan Spark plate reader, and kinase activity or inhibition was calculated relative to controls.

To extend the analysis to other clinically relevant kinases, the inhibitory activity of imatinib, compound **8**, and compound **9** was further tested against PDGFRα (D842Y) and CSF-1R using Chemi-Verse kinase assays (Cambridge Biosciences). Reactions were set up under the supplier’s recommended conditions, with recombinant kinases (5 ng/μL final), peptide substrates, and ATP in kinase assay buffer. Test inhibitors or vehicle were added, and reactions were incubated at 30 °C for 45 min. ADP formation was quantified with ADP-Glo detection, and percent inhibition was determined relative to DMSO controls.

#### Molecular Modeling

A cryo-EM-based model of P-gp bound to encequidar (PDB ID: 7O9W) was used for the molecular modeling study. The molecular docking studies were performed in YASARA Structure where an AutoDock Vina simulation was employed following the designation of a local binding site as described above. The generated poses were subsequently visualized using BIOVIA Discovery Studio Visualizer where 2D and 3D binding interaction maps were generated. Where 3D receptor–ligand interactions are examined, only the binding site surface is presented and is color-coded on a scale of hydrophobicity, where blue indicates hydrophilic regions and brown signifies hydrophobic regions.

## Supplementary Material











## Notes

Characterization of compounds NMR, LCMS and HRMS; LCMS methods and purity data of compounds; Cytotoxicity dose–response curves and kinase activity data; The *m*/*z* of precursor, product ion and collision energy used in the accumulation assay; Comparison of intracellular accumulation of imatinib and compounds 8 and 9 in K562 wild-type and K562/DOX cells (PDF)
Molecular modeling poses of compounds and imatinib with P-gp (model in Figure 2A) (PDB)
Molecular modeling poses of compounds and imatinib with P-gp (model in Figure 2B) (PDB)
Molecular modeling poses of compounds and imatinib with P-gp (model in Table S1) (ZIP)
Molecular formula strings (CSV)

## ABBREVIATIONS USED

Alloc: (allyloxy)­carbonyl
BCR-ABL1: breakpoint cluster region-Abelson 1
CLL: chronic lymphocytic leukemia
cryo-EM: cryo-electron microscopy
CSF-1R: colony stimulating factor 1 receptor
DCM: dichloromethane
DMSO: dimethyl sulfoxide
DOX: doxorubicin
ESI-MS: electrospray ionization mass spectrometry
HCl: hydrochloric acid
LC_50_: lethal concentration 50%
LCMS: liquid chromatography-mass spectrometry
MTT: 3-(4,5-dimethylthiazol-2-yl)-2,5-diphenylte-trazolium bromide
PDGFRα (D842Y): platelet-derived growth factor receptor alpha (D842Y mutation)
Pd­(PPh_3_): palladium­(0) triphenylphosphine
THP: tetrahydropyr-an-2-yl
