# Supplementary material for: Design, Synthesis, and Biological Evaluation of Efflux-Resistant Imatinib Derivatives
Source: J Med Chem. 2025 Oct 25;68(21):22619–32. doi: 10.1021/acs.jmedchem.5c01596 (PMC12621183; doi:10.1021/acs.jmedchem.5c01596)
Supplement: Supplementary file 1 [file jm5c01596_si_001.pdf]

Supporting Information

Design, synthesis, and biological evaluation of efflux-resistant imatinib derivatives

Madiha M. Chowdhury[1], Priantha Pretheshan[1], Nasima S. Chowdhury[1], Paolo Andriollo[1], Ajit J. Shah[2], Ben Forbes[1], Chris Pepper[3]*, Khondaker Miraz Rahman[1]*

[1]Institute of Pharmaceutical Science, King's College London, London, SE1 9NH, U.K.

[2]Department of Natural Sciences, University of Middlesex, The Burroughs, Hendon, London NW4 4BT, U.K.

[3]Brighton and Sussex Medical School, University of Brighton and University of Sussex, Brighton, BN1 9PX, U.K.

Corresponding authors.

KMR: E-mail: k.miraz.rahman@kcl.ac.uk. Phone: +44 (0) 207 8481891

CP: E-mail : C.Pepper@bsms.ac.uk. Phone: +44 (0) 1273 678644

Content:

Table of Contents

Interacting residues with P-gp 2

Cytotoxicity dose-response curves for K562 wild-type cells 3

Kinase Inhibition Data 5

Cytotoxicity curves in K562 wild-type vs K562/DOX 6

Cytotoxicity curves in K562/DOX with and without verapamil 8

Inhibition of PDGFRα (D842Y) kinase activity data 9

The m/z of precursor, product ion and collision energy used for detection of each compound tested in the accumulation assay 10

Comparison of intracellular acccumulation of imatinib and compounds 8 and 9 in K562 wild-type and K562/DOX cells 11

NMR and HRMS spectra, LCMS traces 13

Percentage purity for final compounds as determined by HPLC 30

Interacting residues with P-gp

Table S1: Table showing interacting residues between P-gp region and imatinib analogues.

R	Compound code	Key interacting hydrophilic residues	Key interacting hydrophobic residues	Common residues with encequidar in inhibitor site
	Imatinib	7	18	12
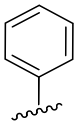	7	2	20	13
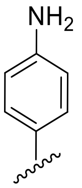	8	6	20	13
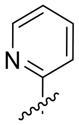	9	7	14	13
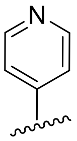	10	3	17	13
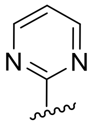	11	6	18	14
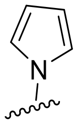	12	5	16	13
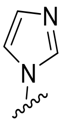	13	4	13	12
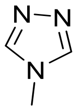	14	5	17	14

Cytotoxicity dose-response curves for K562 wild-type cells

Figure S1: Cytotoxicity dose-response curves for imatinib and compounds 7-11 in K562 wild-type cells. Curves were plotted as the mean (± SD) of three separate experiments. Reported LC_{50} values correspond to mean LC_{50} obtained from at least independent repeat experiments with the SD.

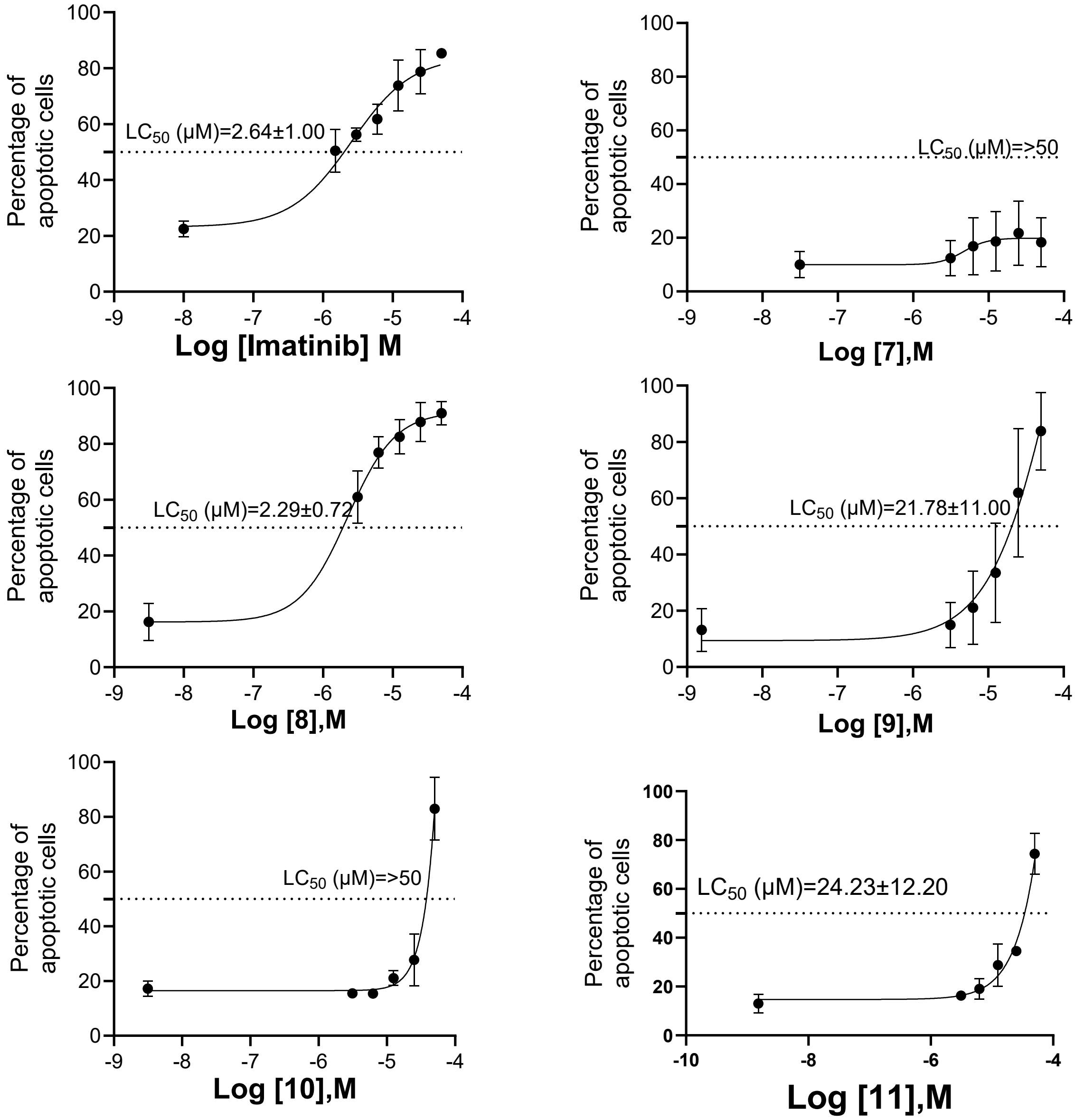

Figure S2: Cytotoxicity dose-response curves for compounds 12-14 in K562 wild-type cell line. Curves were plotted as the mean (± SD) of three separate experiments. Reported LC_{50} values correspond to mean LC_{50} obtained from at least independent repeat experiments with the SD.

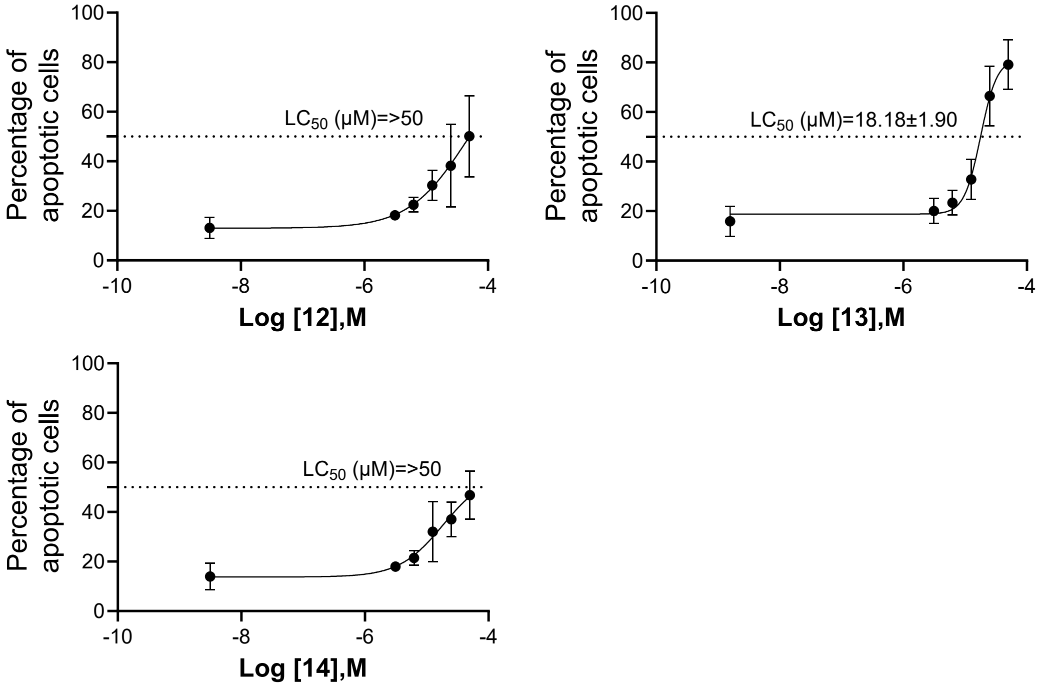

Kinase Inhibition Data

Figure S3: Percentage kinase inhibition by imatinib and analogs at 10 µM after 40 minutes of incubation. The bar graph shows mean % kinase inhibition ± SD for each compound (n =3), with individual data points overlaid. Imatinib showed the highest inhibition (52.62%), while analogs displayed variable inhibitory effects ranging from 26.65% to 47.43%. Chemical structures of each analog are shown below the corresponding bars.

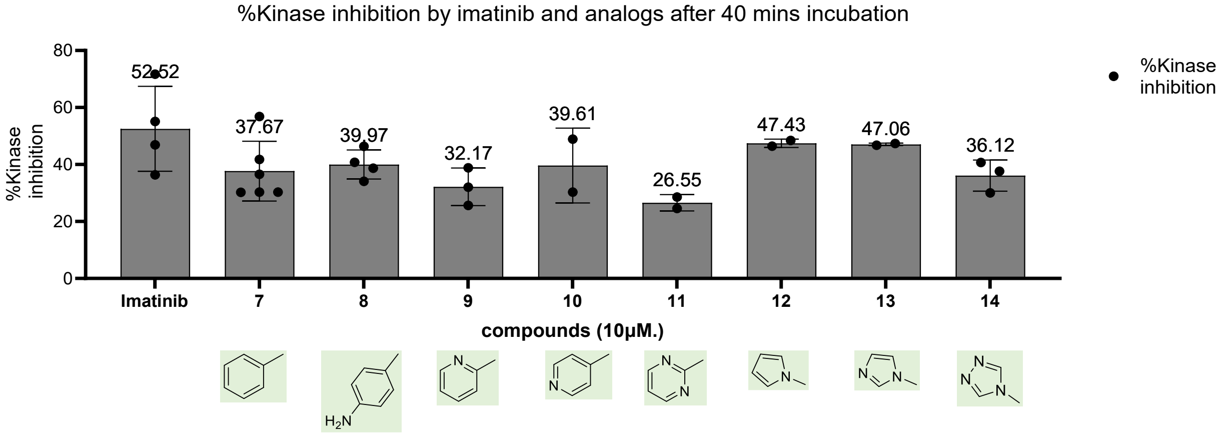

Cytotoxicity curves in K562 wild-type vs K562/DOX

Figure S4: Cytotoxicity dose-response curves for compounds 8-11 in in K562/DOX cell line and K562 wild-type. Curves were plotted as the mean (± SD) of three separate experiments. Reported LC_{50} values correspond to mean LC_{50} obtained from at least independent repeat experiments with the SD.

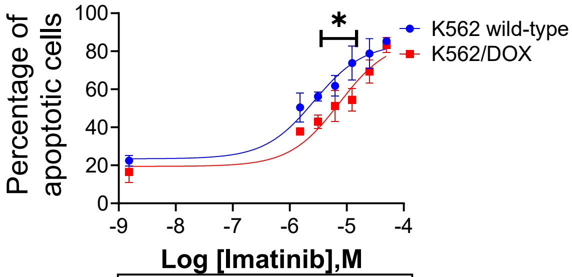

LC_{50} (μM)=
K562 wild-type=2.64±1.00
K562/DOX=6.65±1.30

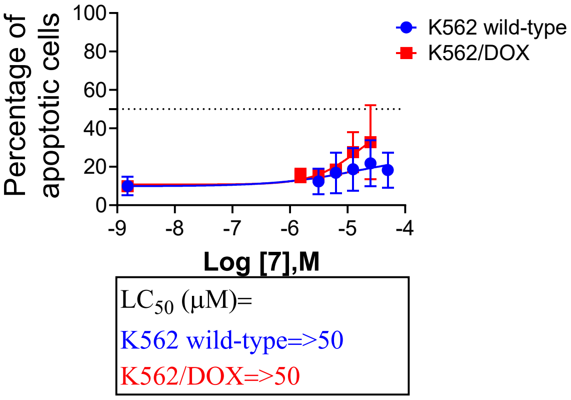

LC_{50} (μM)=
K562 wild-type=>50
K562/DOX=>50

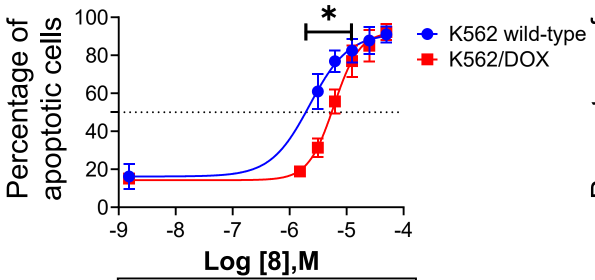

LC_{50} (μM)=
K562 wild-type=2.29±0.72
K562/DOX=5.29±0.71

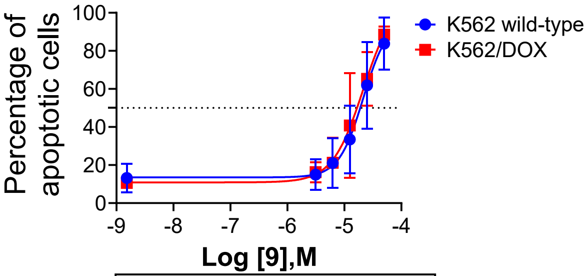

LC_{50} (μM)=
K562 wild-type=21.78±11.05
K562/DOX=20.26±10.30

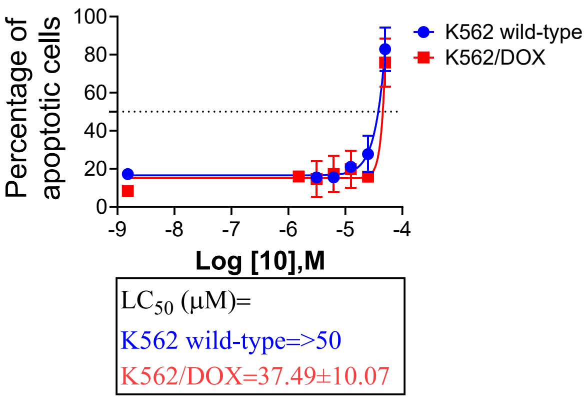

LC_{50} (μM)=
K562 wild-type=>50
K562/DOX=37.49±10.07

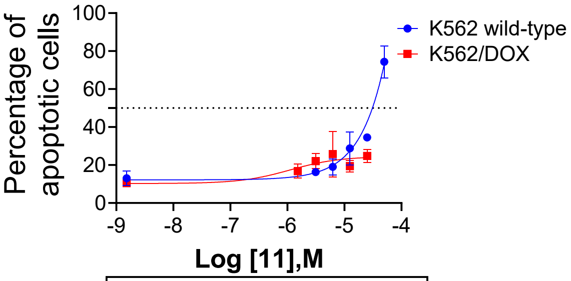

LC_{50} (μM)=
K562 wild-type=24.23±12.20
K562/DOX=>50

Figure S5: Cytotoxicity dose-response curves for compounds 12-14 in K562/DOX cell line and K562 wild-type. Curves were plotted as the mean (± SD) of three separate experiments. Reported LC_{50} values correspond to mean LC_{50} obtained from at least independent repeat experiments with the SD.

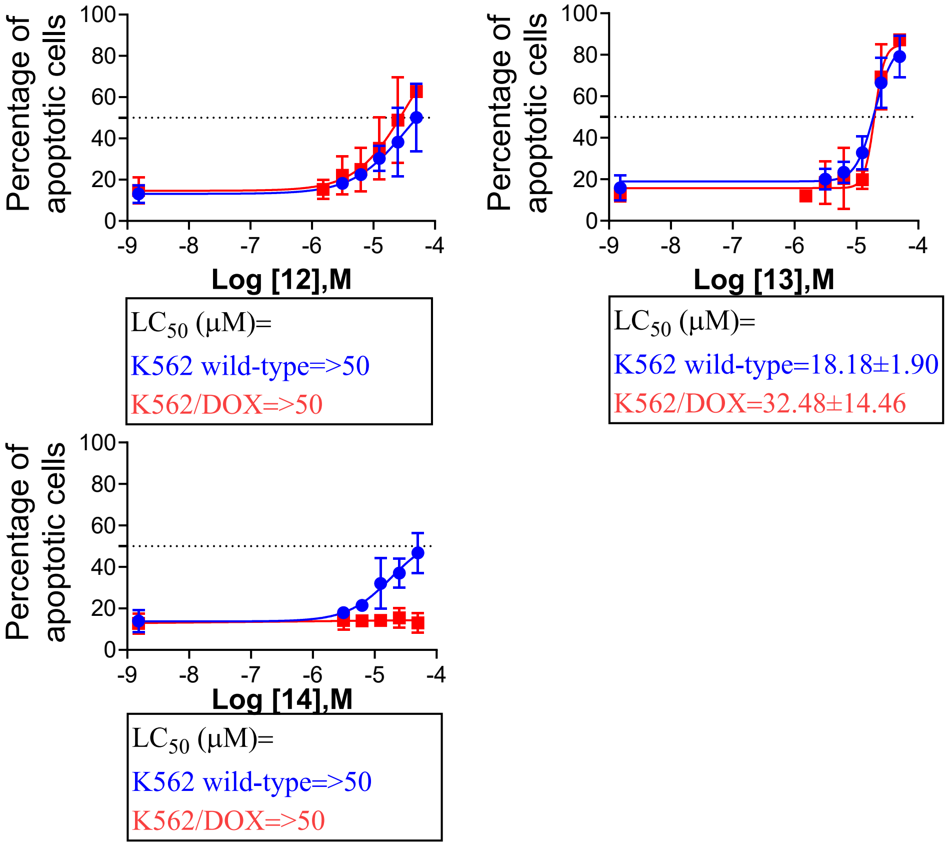

Cytotoxicity curves in K562/DOX with and without verapamil

Figure S6: Cytotoxicity dose-response curves for compounds 9 and 13 in K562/DOX cell line with and without verapamil. Curves were plotted as the mean (± SD) of three separate experiments. Reported LC_{50} values correspond to mean LC_{50} obtained from at least independent repeat experiments with the SD.

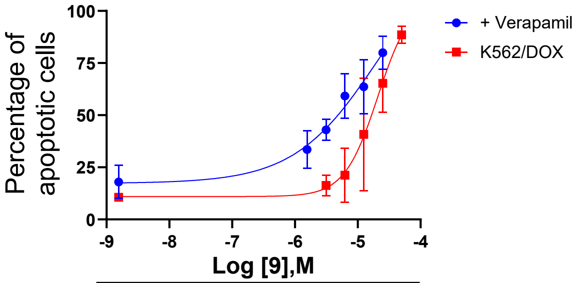

LC_{50} (μM.)
With verapamil=5.00±2.27
K562/DOX=20.26±10.30

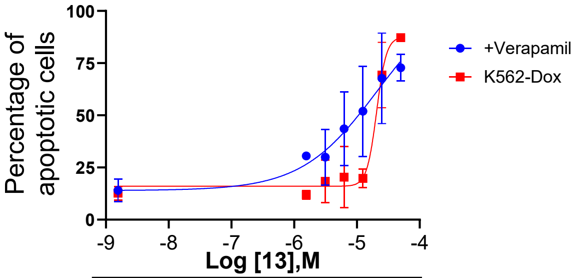

LC_{50} (μM.)
With verapamil=19.09±11.21
K562/DOX=32.48±14.46

Inhibition of PDGFRα (D842Y) kinase activity data

Figure S7a: Inhibition of PDGFRα (D842Y) kinase activity by Imatinib and compounds 8 and 9. PDGFRα (D842Y) kinase activity was measured in the presence of increasing concentrations of Imatinib and Compounds 8 and 9. The "Blank" value was subtracted from all other values. Results are expressed as the percent of control (kinase activity in the absence of inhibitor, set at 100%).

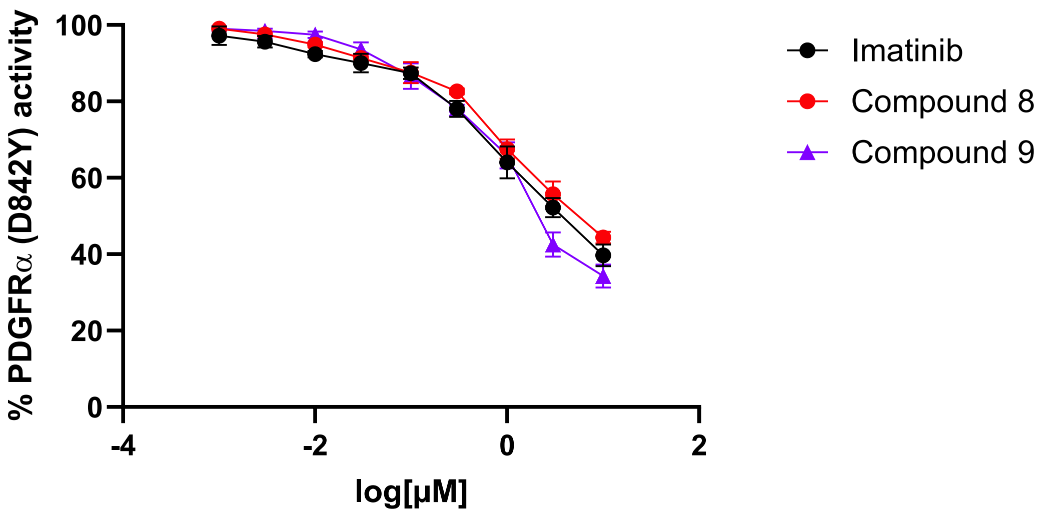

Figure S7b: ***Inhibition of CSF-1R kinase activity by Imatinib and compounds 8 and 9.*** **CSF-1R kinase activity was measured in the presence of increasing concentrations of Imatinib and Compounds 8 and 9. The "Blank" value was subtracted from all other values. Results are expressed as the percent of control (kinase activity in the absence of inhibitor, set at 100%).**

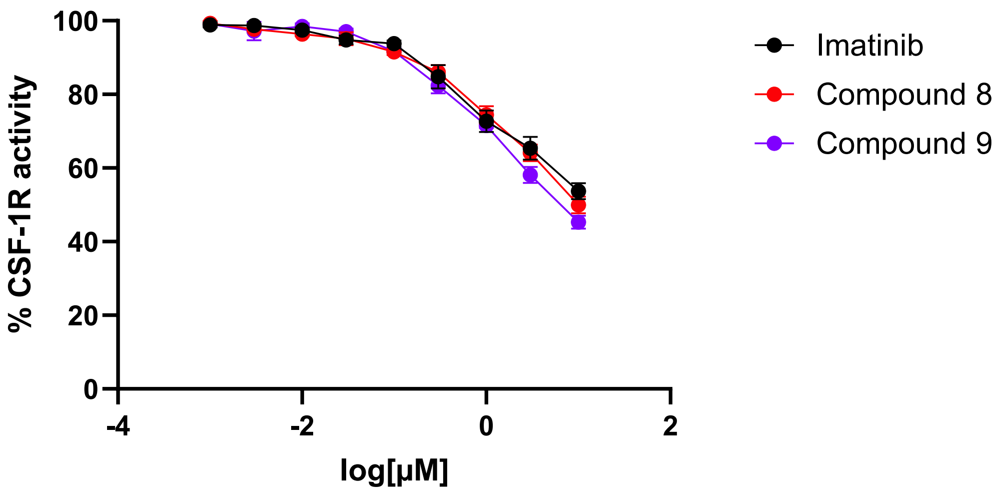

The m/z of precursor, product ion and collision energy used for detection of each compound tested in the accumulation assay

Table S2: Table showing the m/z of the precursor, product ion and collision energy used for the detection of each compound tested in the accumulation assay

Analyte	Precursor *m/z*	Product *m/z*	Collision energy (eV)
imatinib	494	394*	-28
		217	-25
		247	-45
compound **8**	579.7	394	-31
		293	-29
		264*	-48
Compound **9**	556.7	394*	-32
		222	-45
		297	-30

*Transition used for quantitation (the other two product ions were used as qualifiers)

Comparison of intracellular accumulation of imatinib and compounds 8 and 9 in K562 wild-type and K562/DOX cells

Figure S8a: Comparison of intracellular accumulation of imatinib and compound 8 and imatinib and compound 9 in the P-gp overexpressing K562/DOX cells. Statistical significance of the differences in concentration (n=6) have been assessed using the non-parametric Mann-Whitney test.

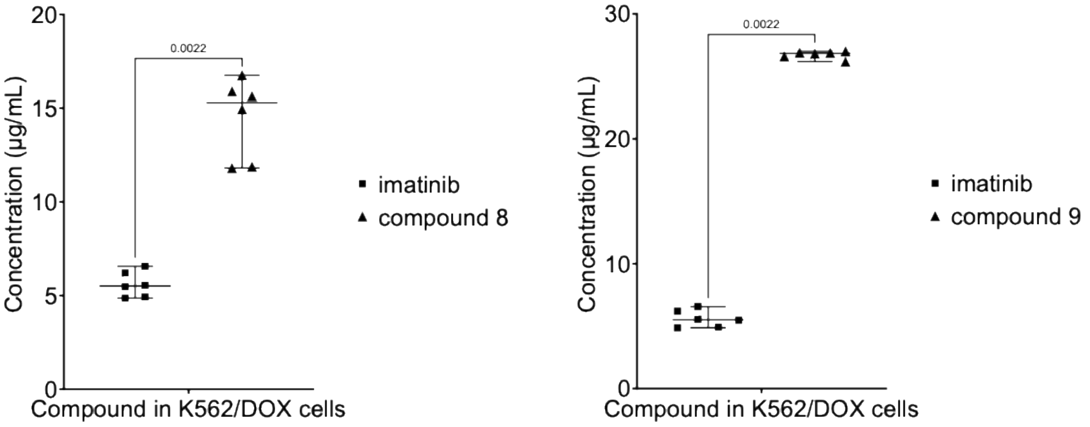

Figure S8b: Comparison of intracellular accumulation of imatinib and compound 8 and imatinib and compound 9 in K562 wild-type cells. Statistical significance of the differences in concentration (n=6) have been assessed using the non-parametric Mann-Whitney test.

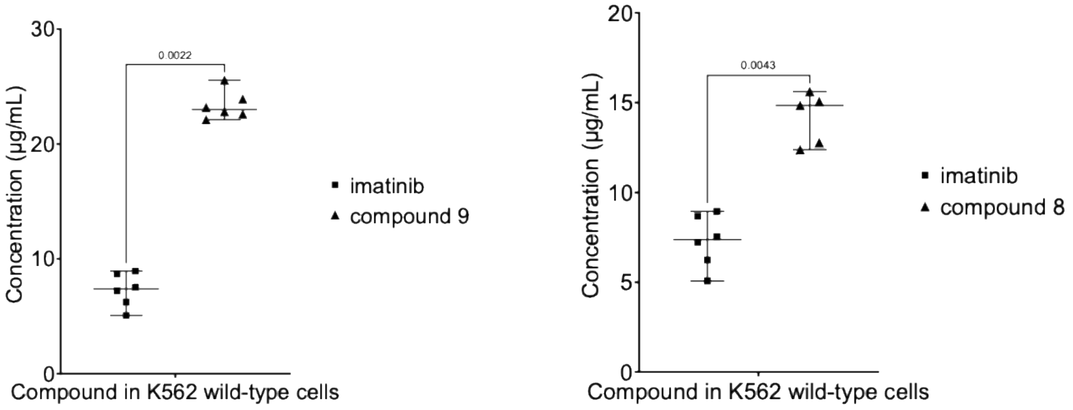

Figure S8c: Comparison of intracellular accumulation of compounds 8 and 9 in K562 wild-type cells and the P-gp overexpressing K562/DOX cells. Statistical significance of the differences in concentration (n=6) have been assessed using the non-parametric Mann-Whitney test.

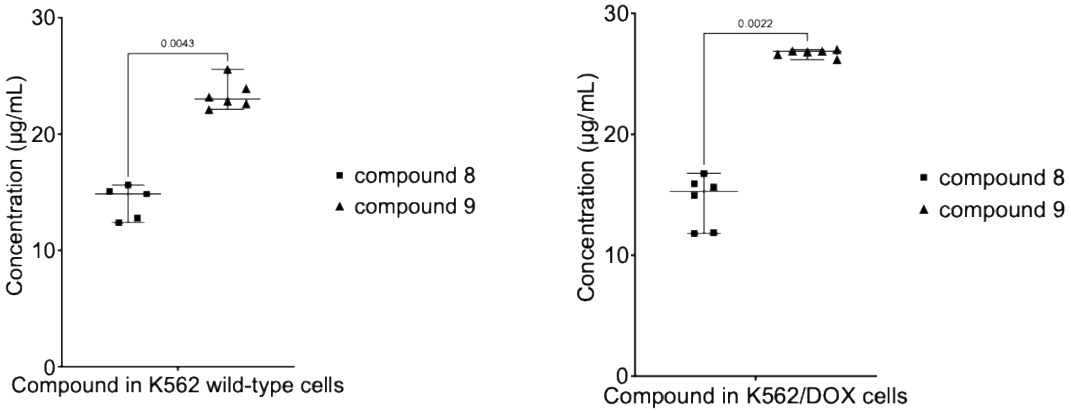

NMR and HRMS spectra, LCMS Traces

Figure S9a: ^{1}H NMR, ^{13}C NMR and HRMS (theoretical result in the bottom and experimental at the top) spectra of **7**

^{1}H NMR (400 MHz, $(CD_3)_2SO$))

4-methyl-3-((4-(pyridin-3-yl) pyrimidin-2-yl) amino) phenyl-4-((4-phenylpiperidin-1-yl)methyl)benzamide

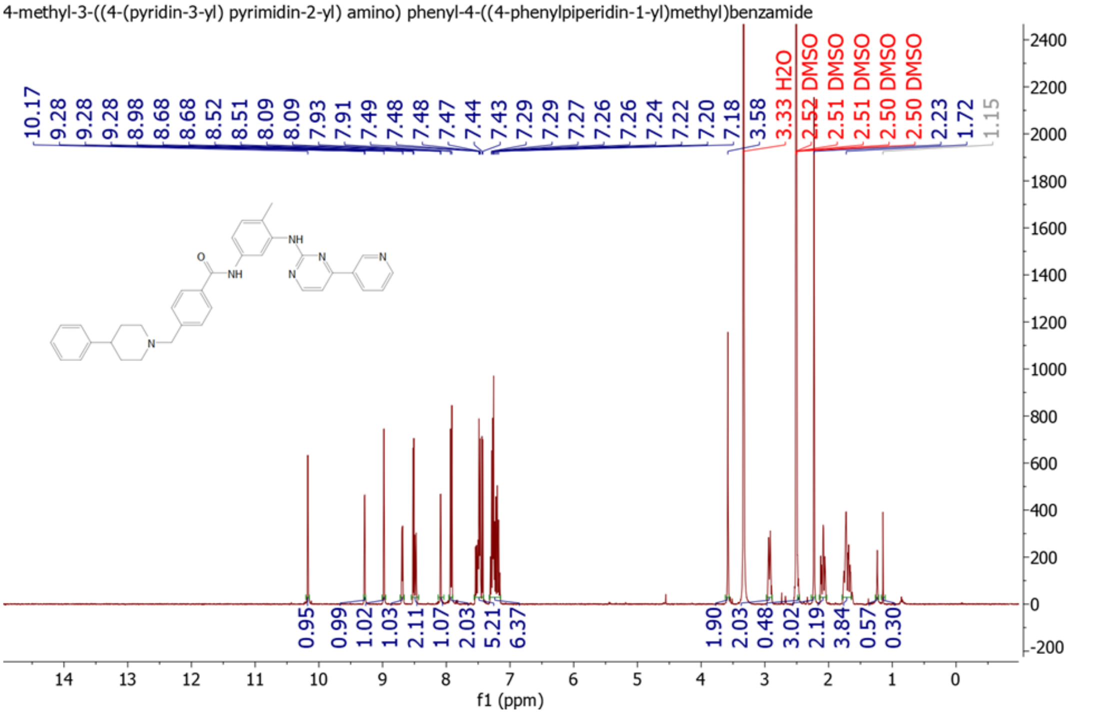

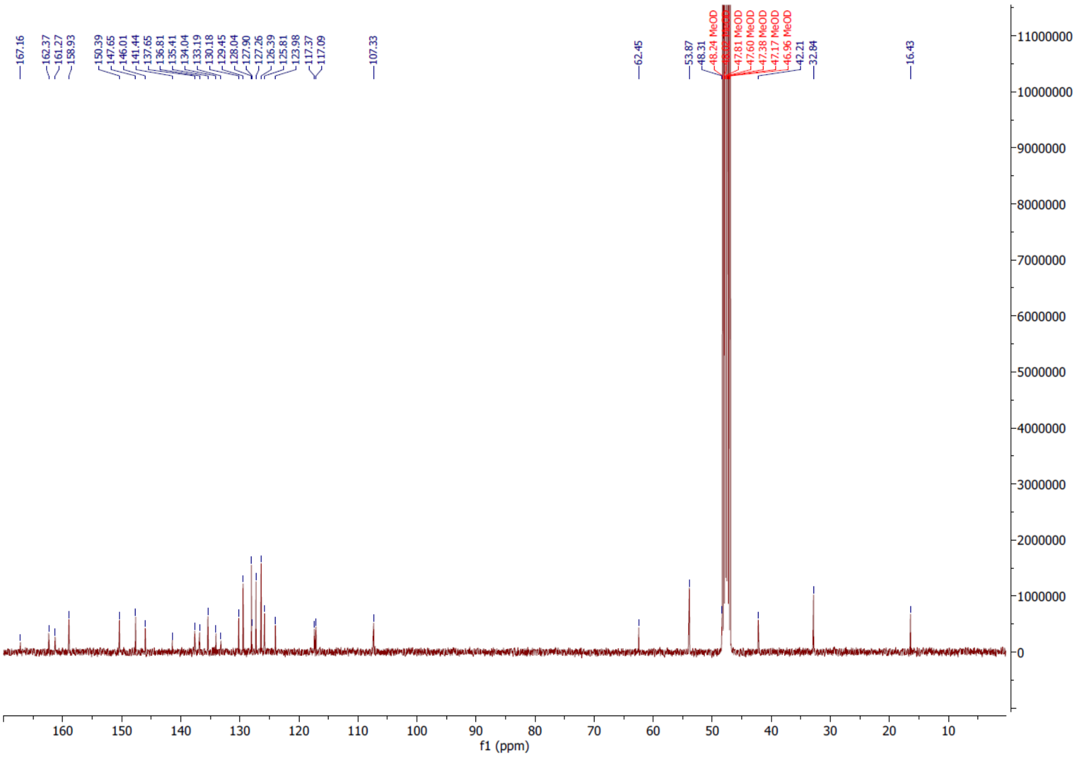

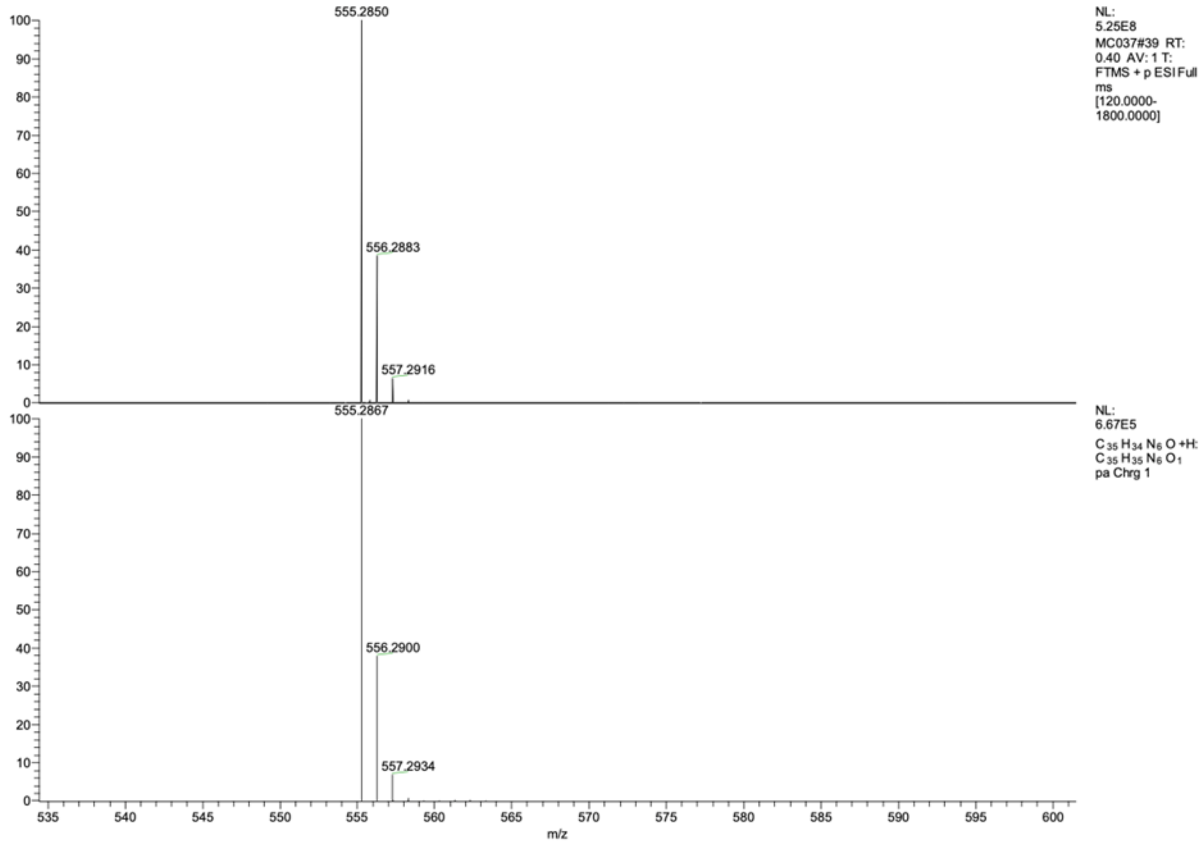

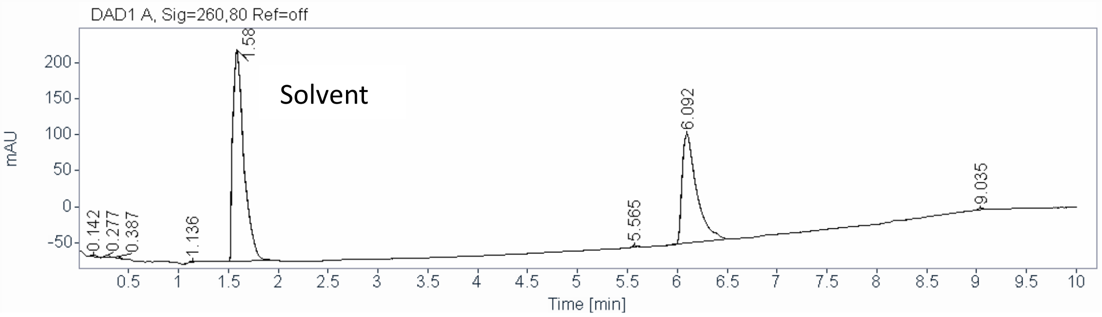

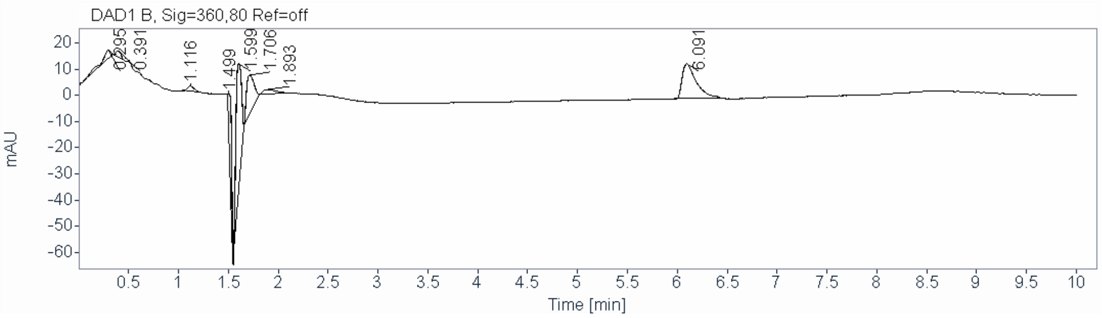

Figure S9b: ^{1}H NMR, ^{13}C NMR and HRMS (theoretical result in the bottom and experimental at the top) spectra of **8**

^{1}H (400MHz, CD_3OD)

4-((4-(4-aminophenyl) piperidin-1-yl) methyl)-N-(4-methyl-3-((4-(yridine-3-yl) pyrimidin-2-yl) amino) phenyl) benzamide

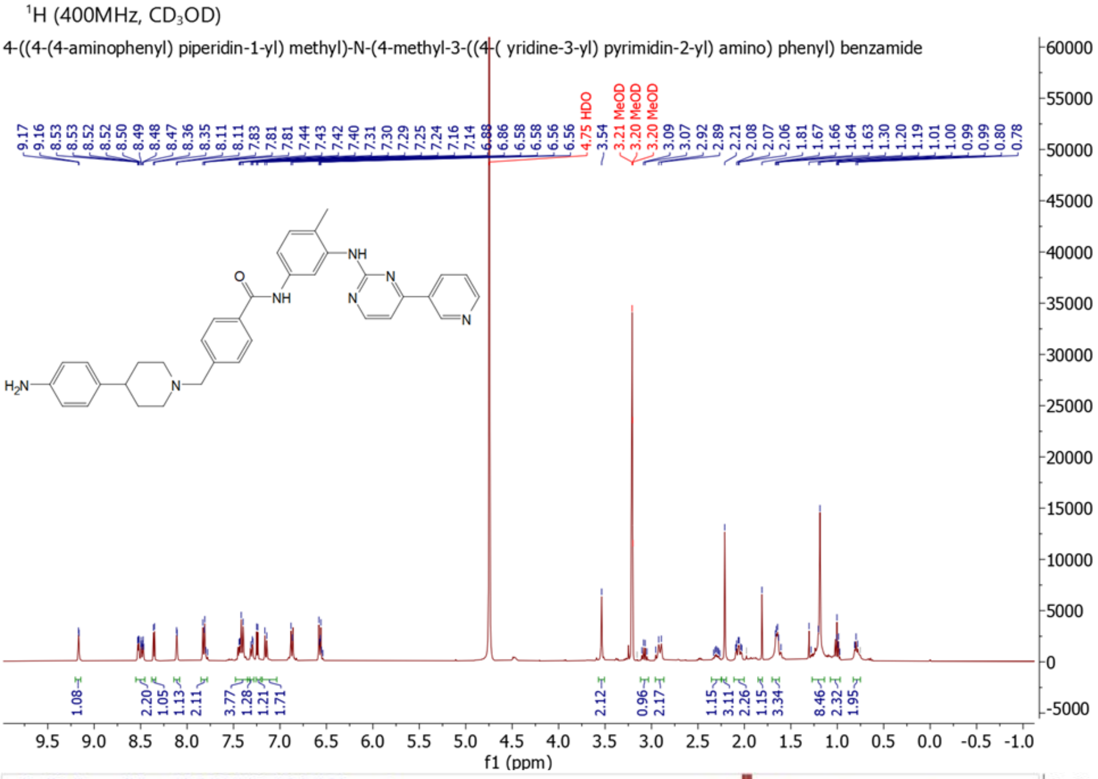

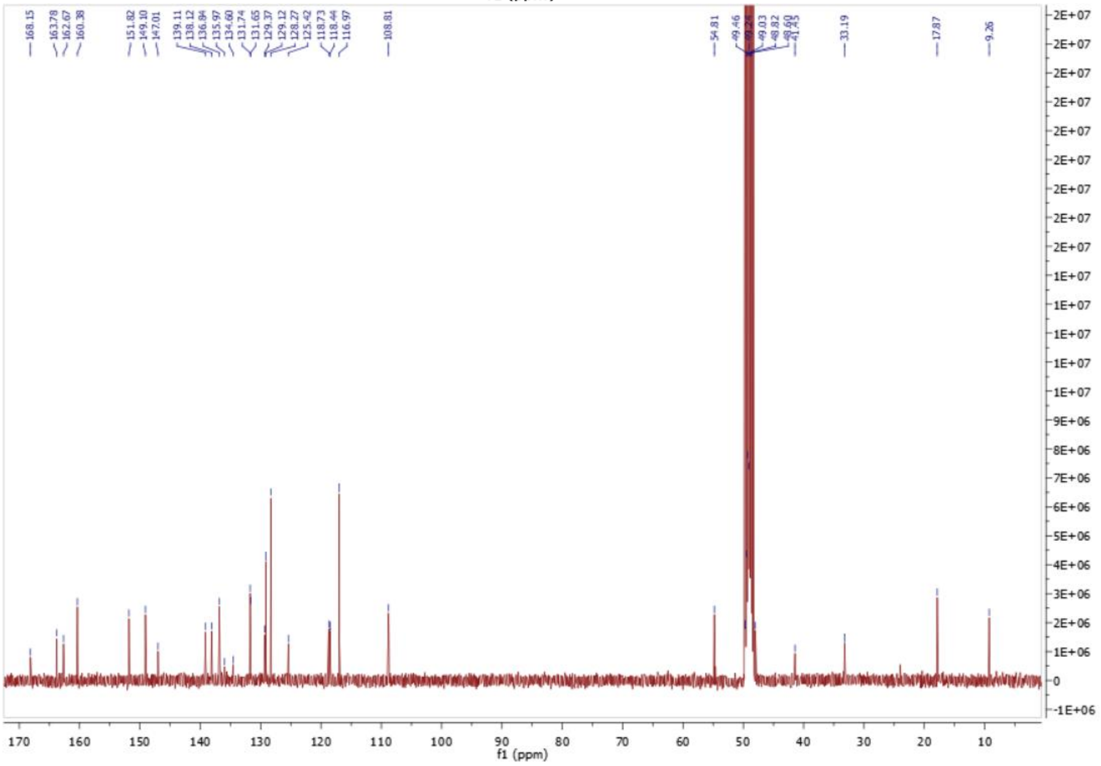

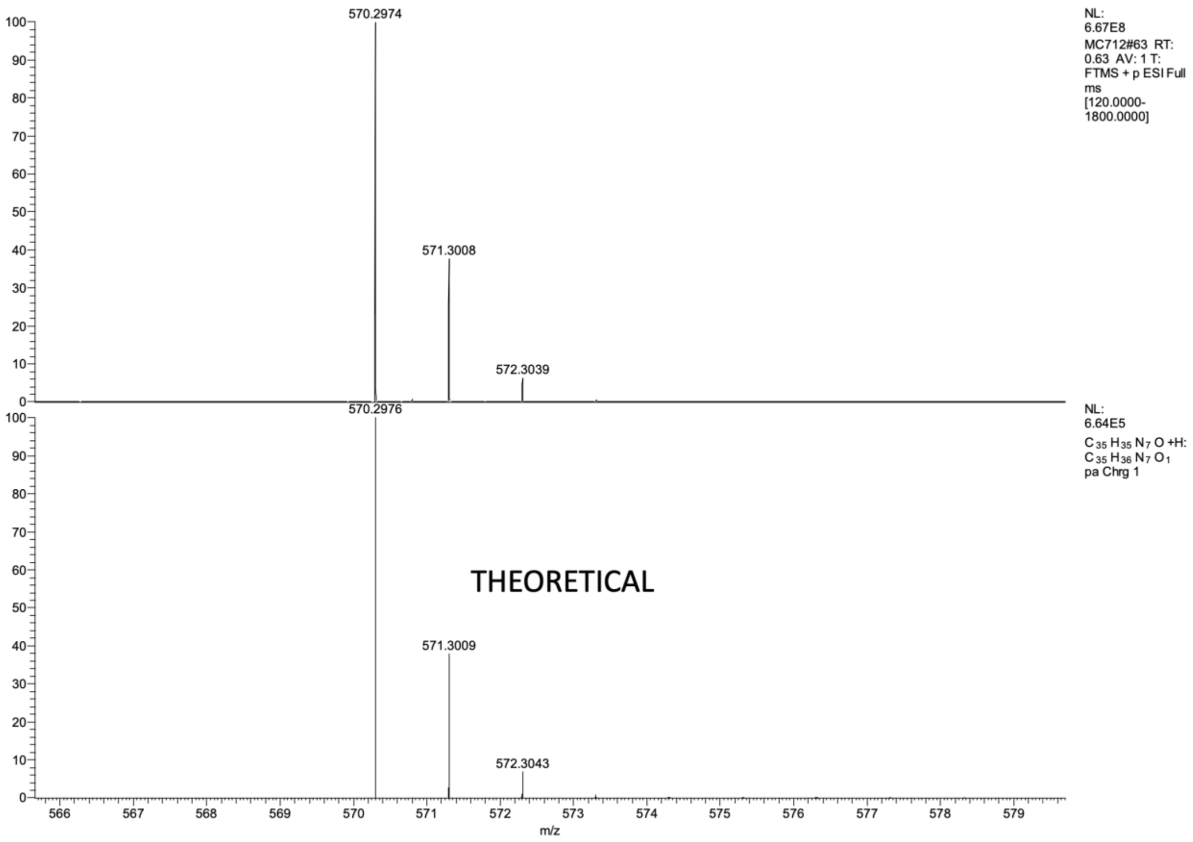
570.2974
571.3008
572.3039
NL:
6.67E8
MC712#63 RT:
0.63 AV: 1 T:
FTMS + p ESI Full
ms
[120.0000-
1800.0000]
570.2976
571.3009
572.3043
NL:
6.64E5
C 35 H 35 N 7 O +H:
C 35 H 36 N 7 O 1
pa Chrg 1
THEORETICAL
m/z

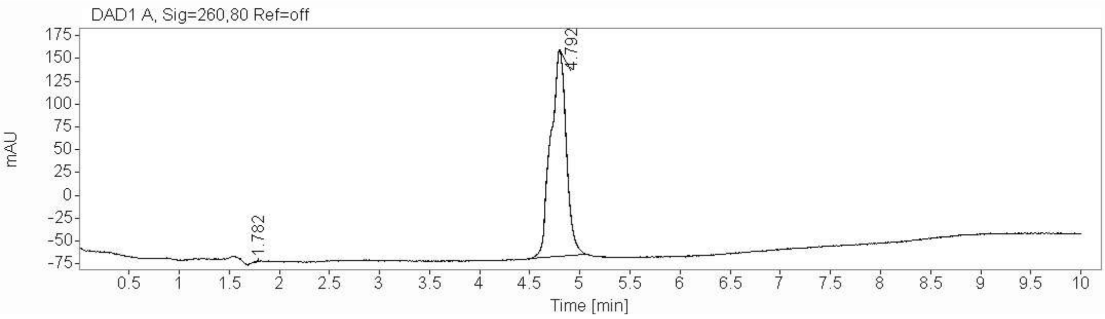
DAD1 A, Sig=260,80 Ref=off
mAU
4.792
1.782
Time [min]

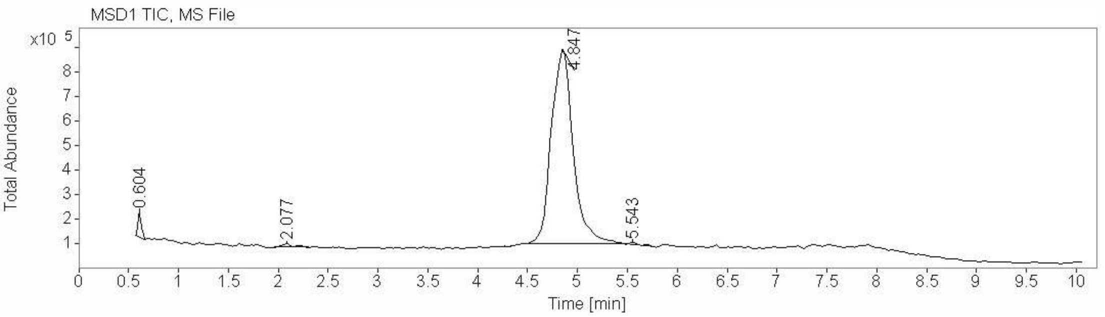
MSD1 TIC, MS File
x10 5
Total Abundance
4.847
0.604
2.077
5.543
Time [min]

Figure S9c: ^{1}H NMR, ^{13}C NMR and HRMS (theoretical result in the bottom and experimental at the top) spectra of **9**

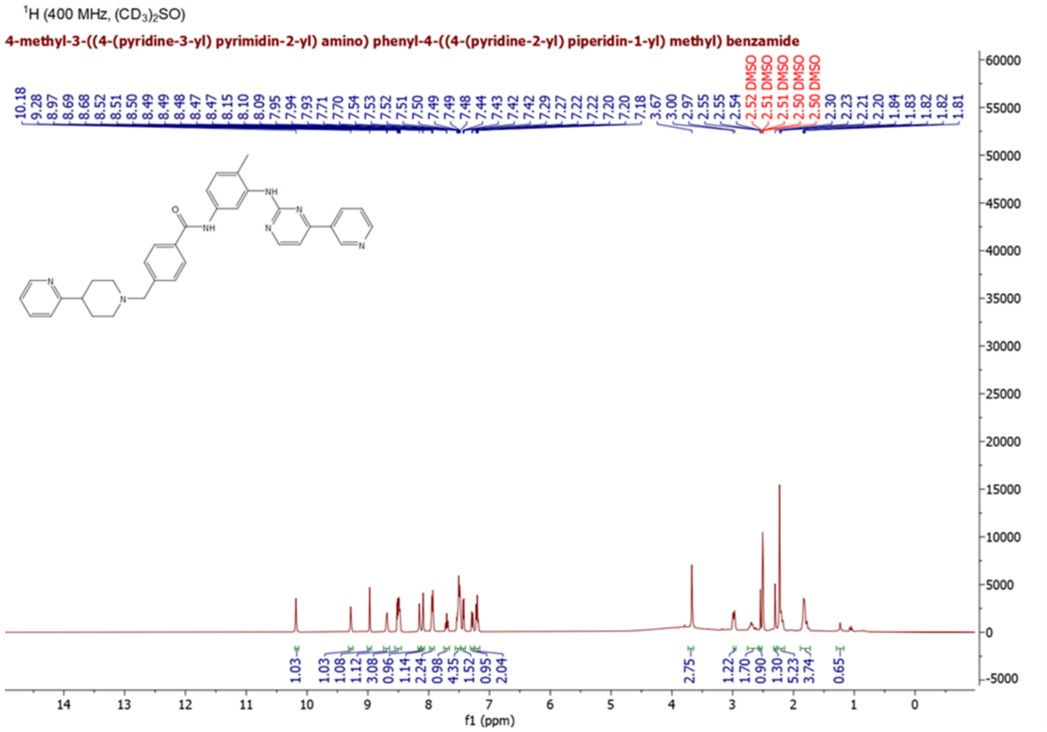

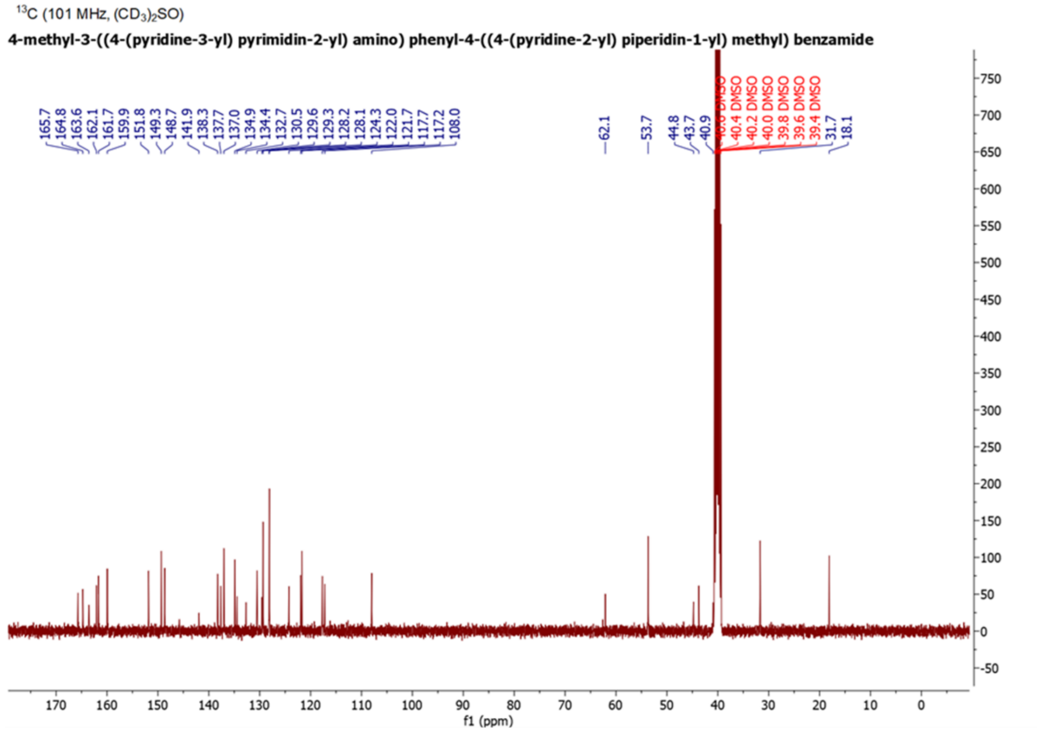

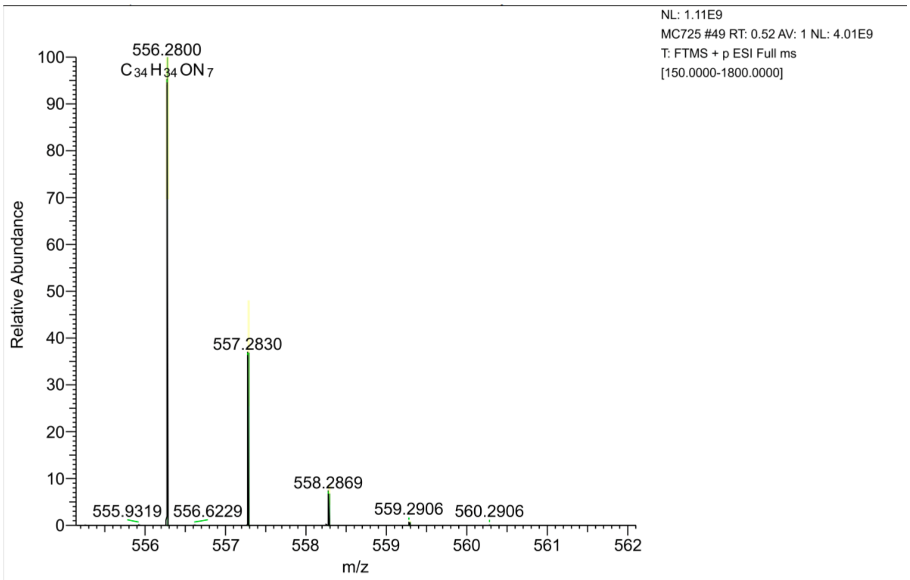

NL: 1.11E9
MC725 #49 RT: 0.52 AV: 1 NL: 4.01E9
T: FTMS + p ESI Full ms
[150.0000-1800.0000]
556.2800
C34 H34 ON7
557.2830
558.2869
555.9319
556.6229
559.2906
560.2906
Relative Abundance
m/z

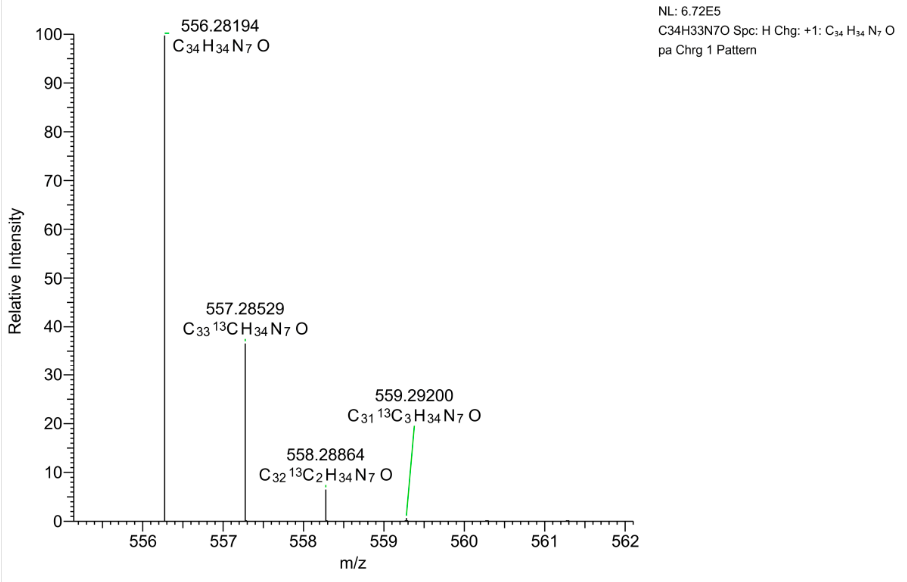

NL: 6.72E5
C34H33N7O Spc: H Chg: +1: C34 H34 N7 O
pa Chrg 1 Pattern
556.28194
C34 H34 N7 O
557.28529
C33 13C H34 N7 O
558.28864
C32 13C2 H34 N7 O
559.29200
C31 13C3 H34 N7 O
Relative Intensity
m/z

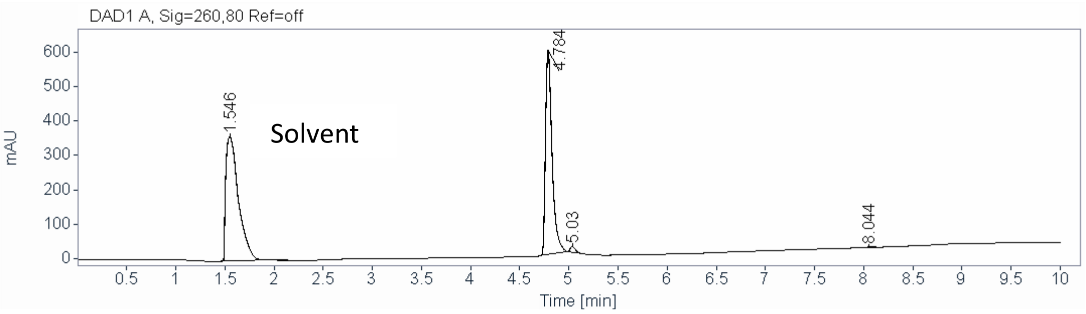

DAD1 A, Sig=260,80 Ref=off
mAU
1.546
Solvent
4.784
5.03
8.044
Time [min]

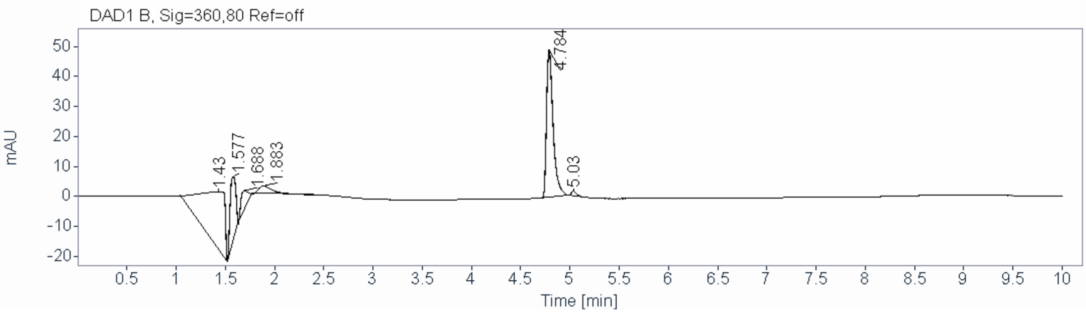

DAD1 B, Sig=360,80 Ref=off
mAU
1.43
1.577
1.688
1.883
4.784
5.03
Time [min]

Figure S9d: ^{1}H NMR, ^{13}C NMR and HRMS (theoretical result in the bottom and experimental at the top) spectra of **10**

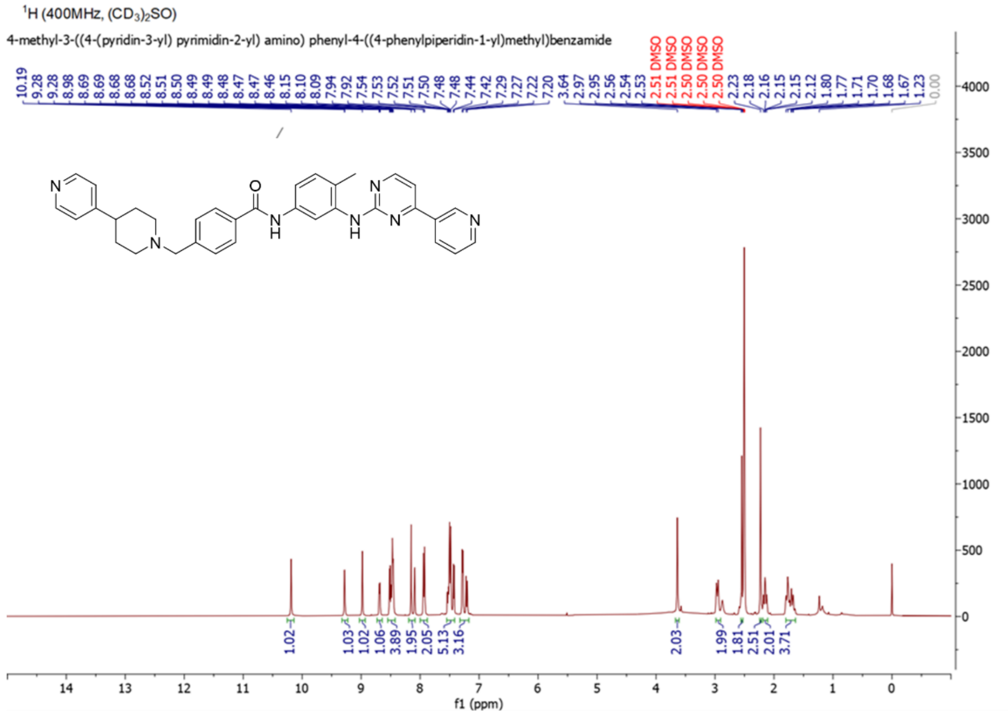

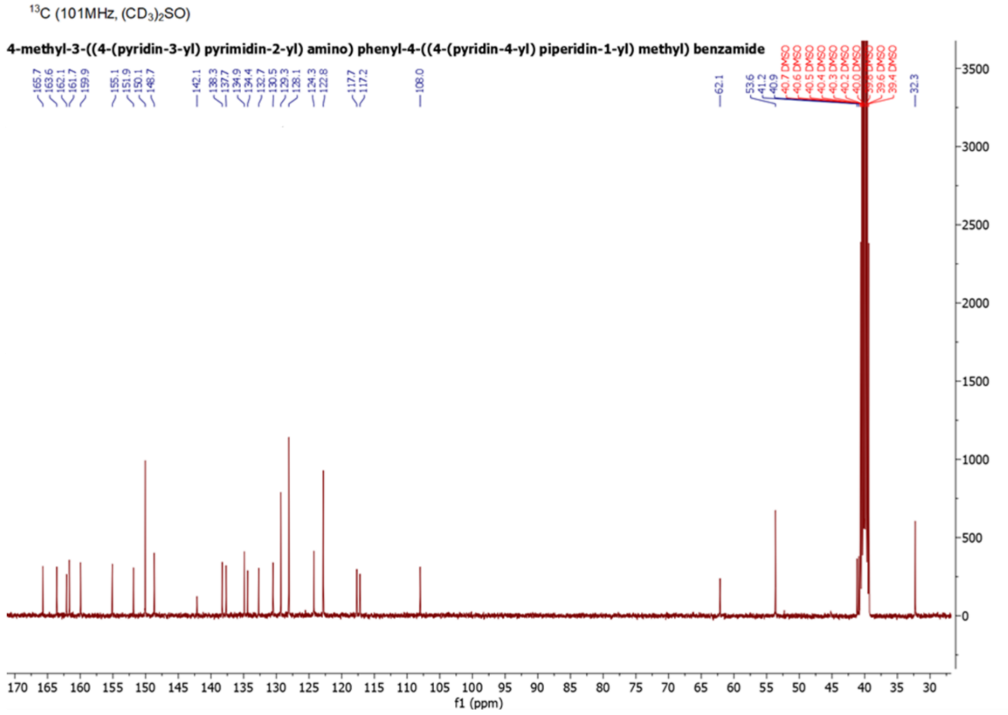

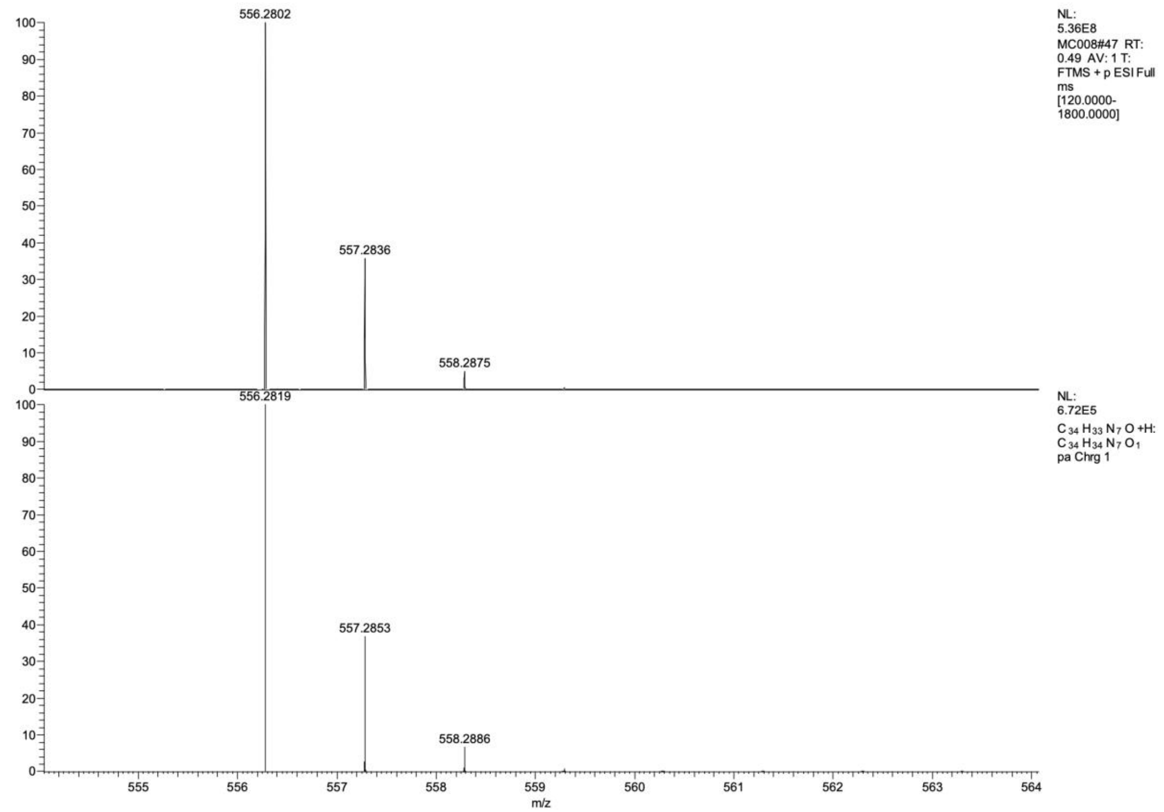

556.2802
557.2836
558.2875
NL: 5.36E8 MC008#47 RT: 0.49 AV: 1 T: FTMS + p ESI Full ms [120.0000-1800.0000]
556.2819
557.2853
558.2886
NL: 6.72E5 C 34 H33 N7 O +H: C 34 H34 N7 O1 pa Chrg 1
m/z

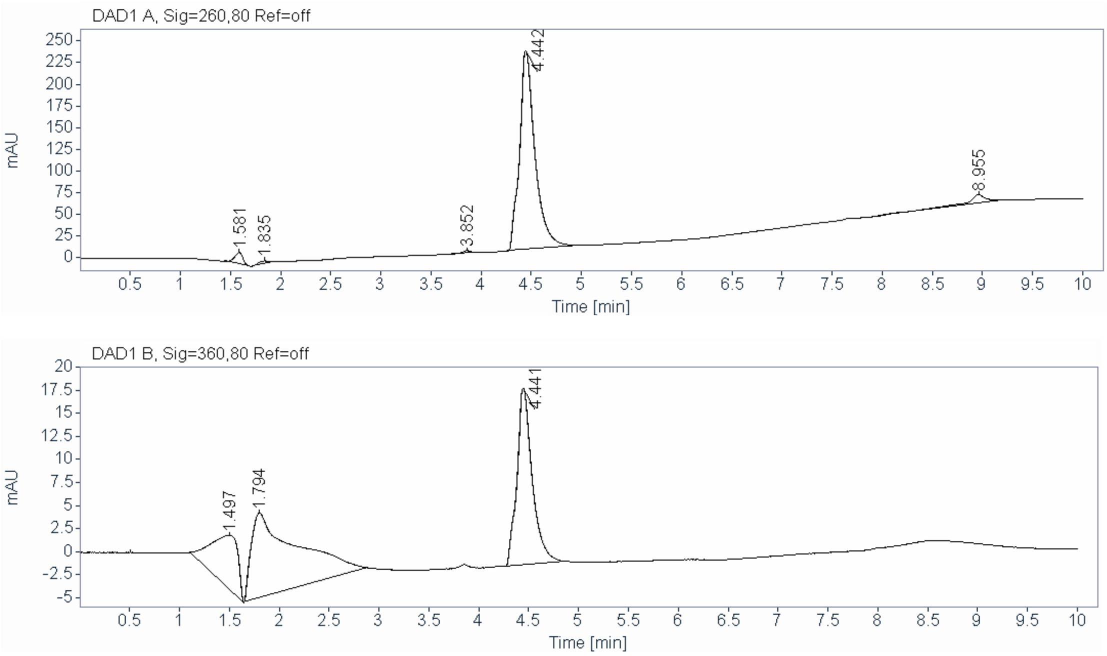

DAD1 A, Sig=260,80 Ref=off
mAU
1.581
1.835
3.852
4.442
8.955
Time [min]
DAD1 B, Sig=360,80 Ref=off
mAU
1.497
1.794
4.441
Time [min]

Figure S9e: ^{1}H NMR, ^{13}C NMR and HRMS (theoretical result in the bottom and experimental at the top) spectra of **11**

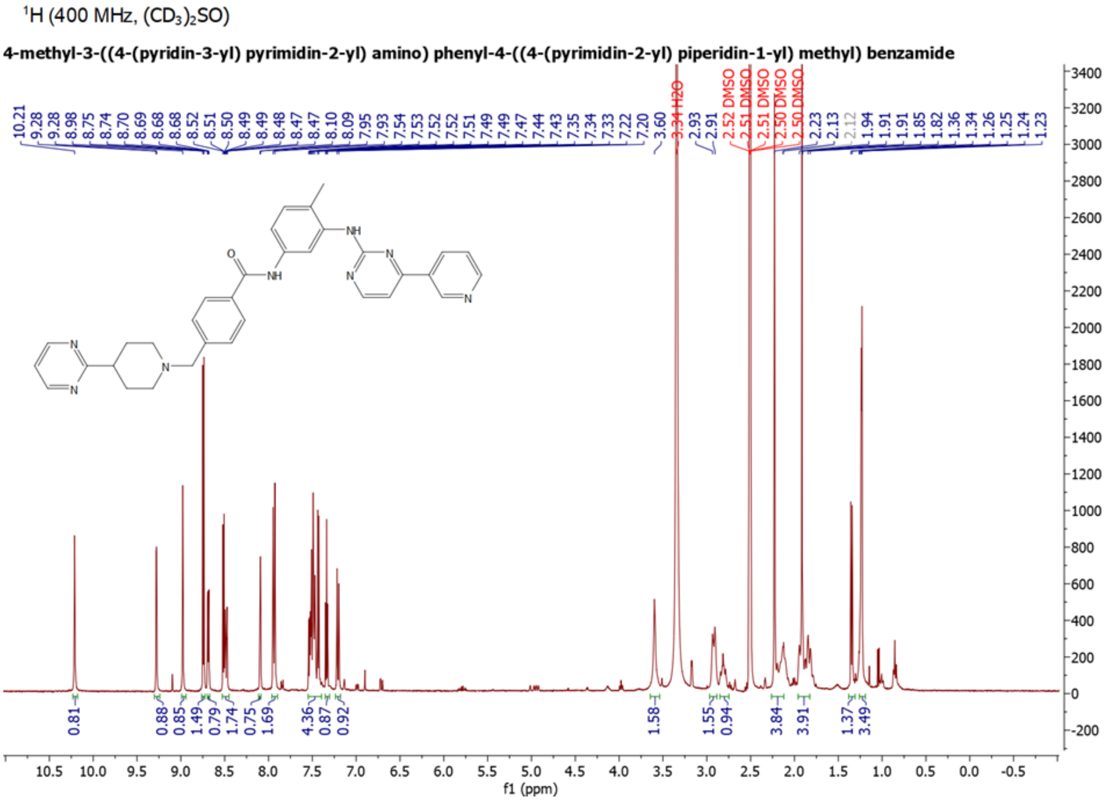

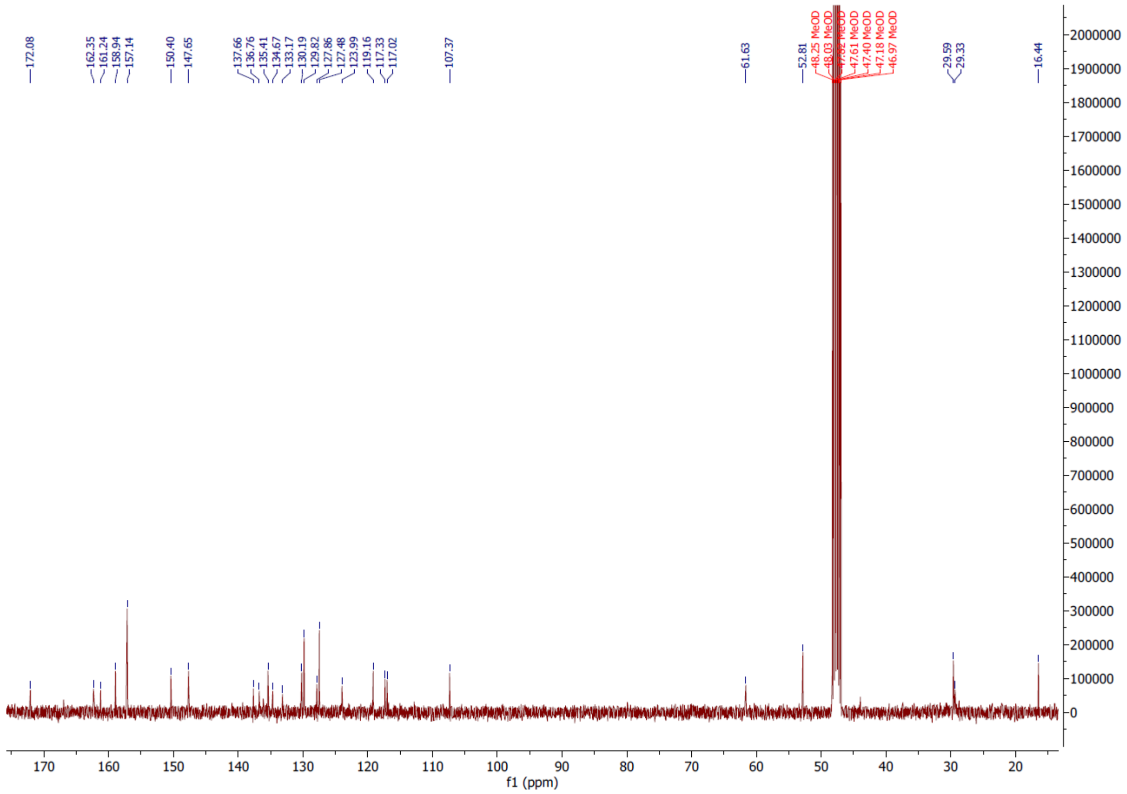

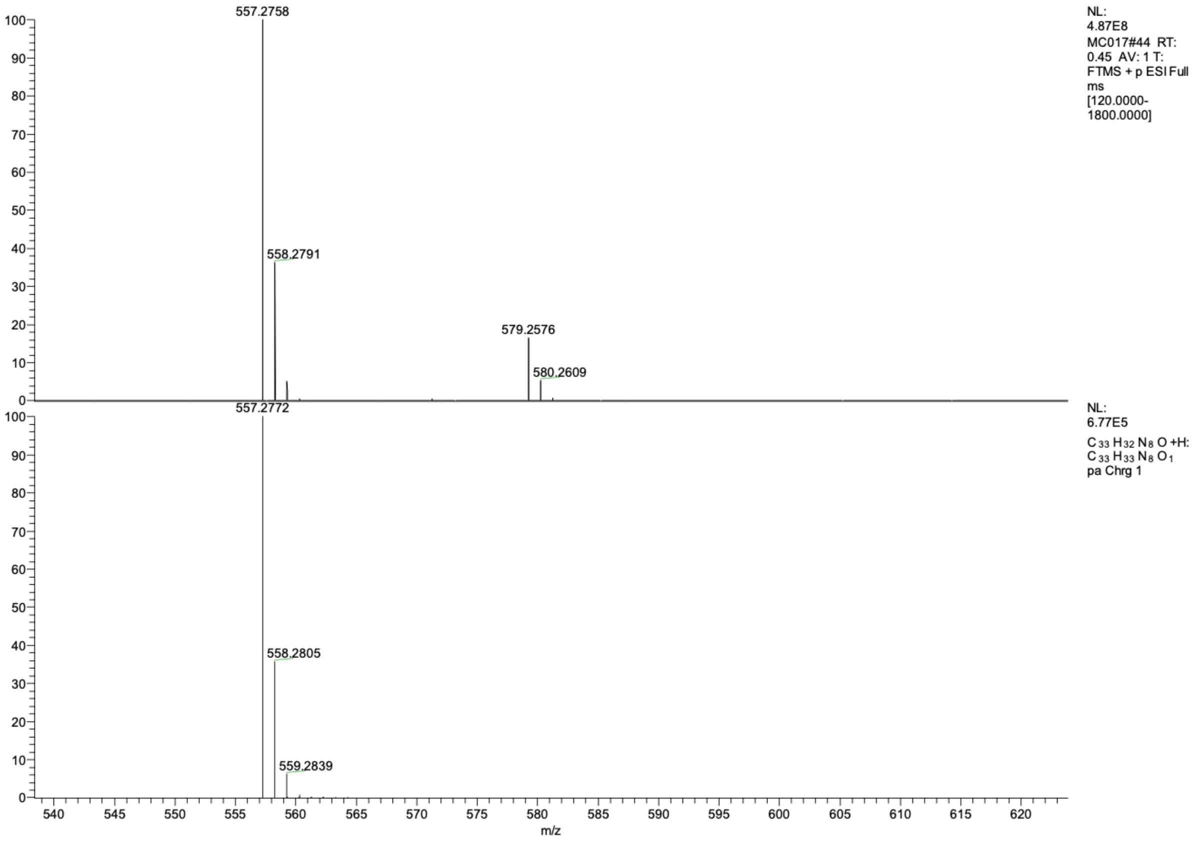

557.2758
NL: 4.87E8 MC017#44 RT: 0.45 AV: 1 T: FTMS + p ESI Full ms [120.0000-1800.0000]
558.2791
579.2576
580.2609
557.2772
NL: 6.77E5
C 33 H 32 N 8 O +H: C 33 H 33 N 8 O 1 pa Chrg 1
558.2805
559.2839
m/z

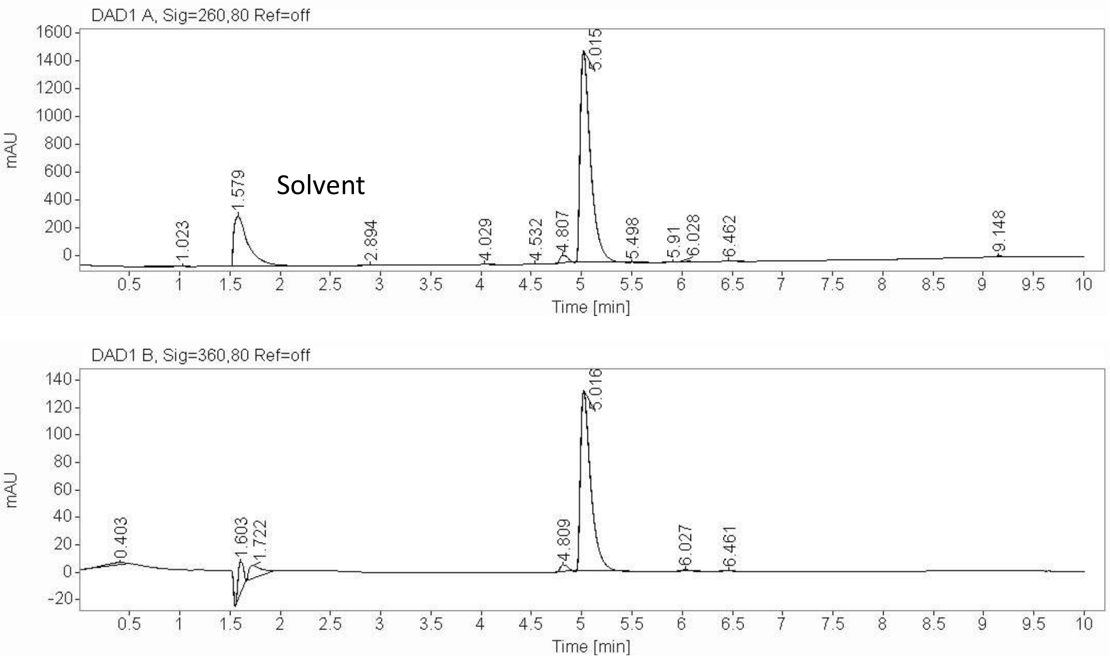

DAD1 A, Sig=260,80 Ref=off
mAU
Solvent
1.023
1.579
2.894
4.029
4.532
4.807
5.015
5.498
5.91
6.028
6.462
9.148
Time [min]
DAD1 B, Sig=360,80 Ref=off
mAU
0.403
1.603
1.722
4.809
5.016
6.027
6.461
Time [min]

Figure S9f: ^{1}H NMR, ^{13}C NMR and HRMS (theoretical result in the bottom and experimental at the top) spectra of **12**

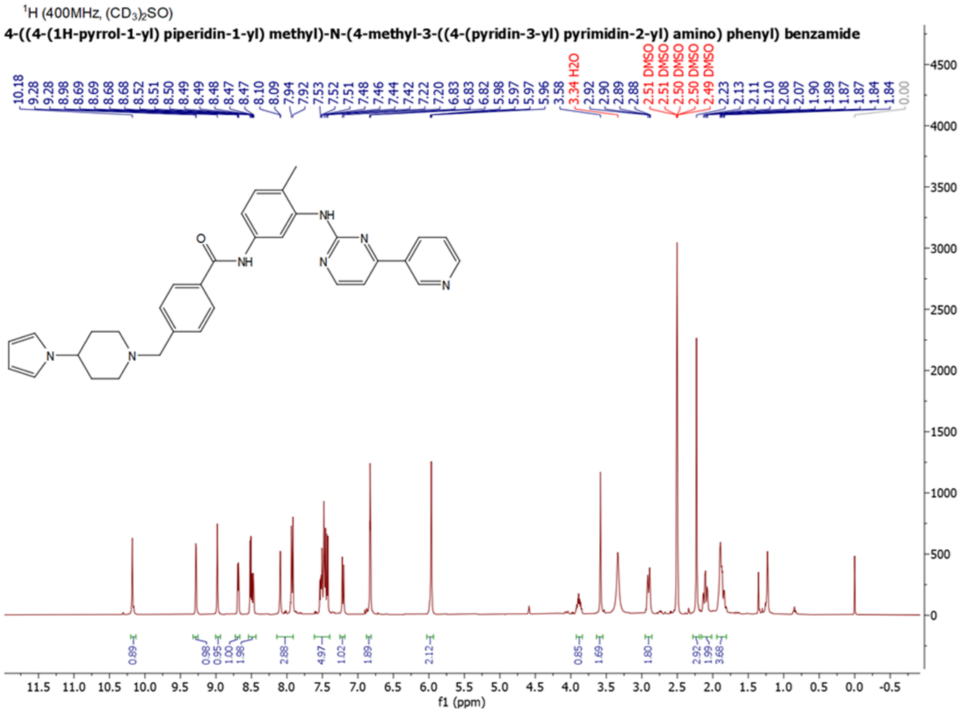

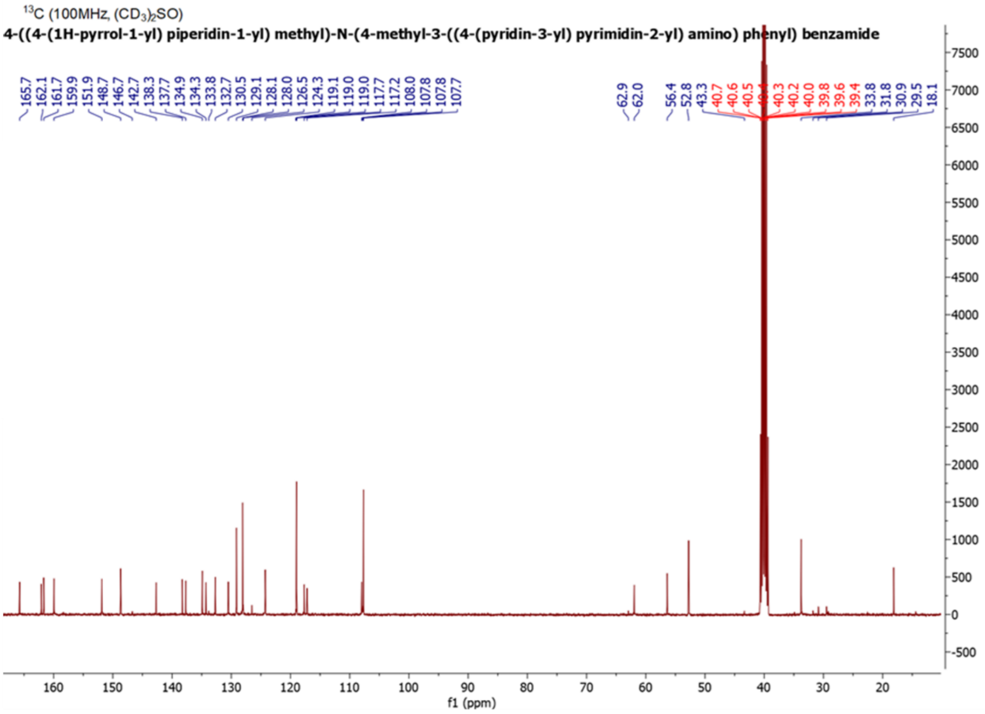

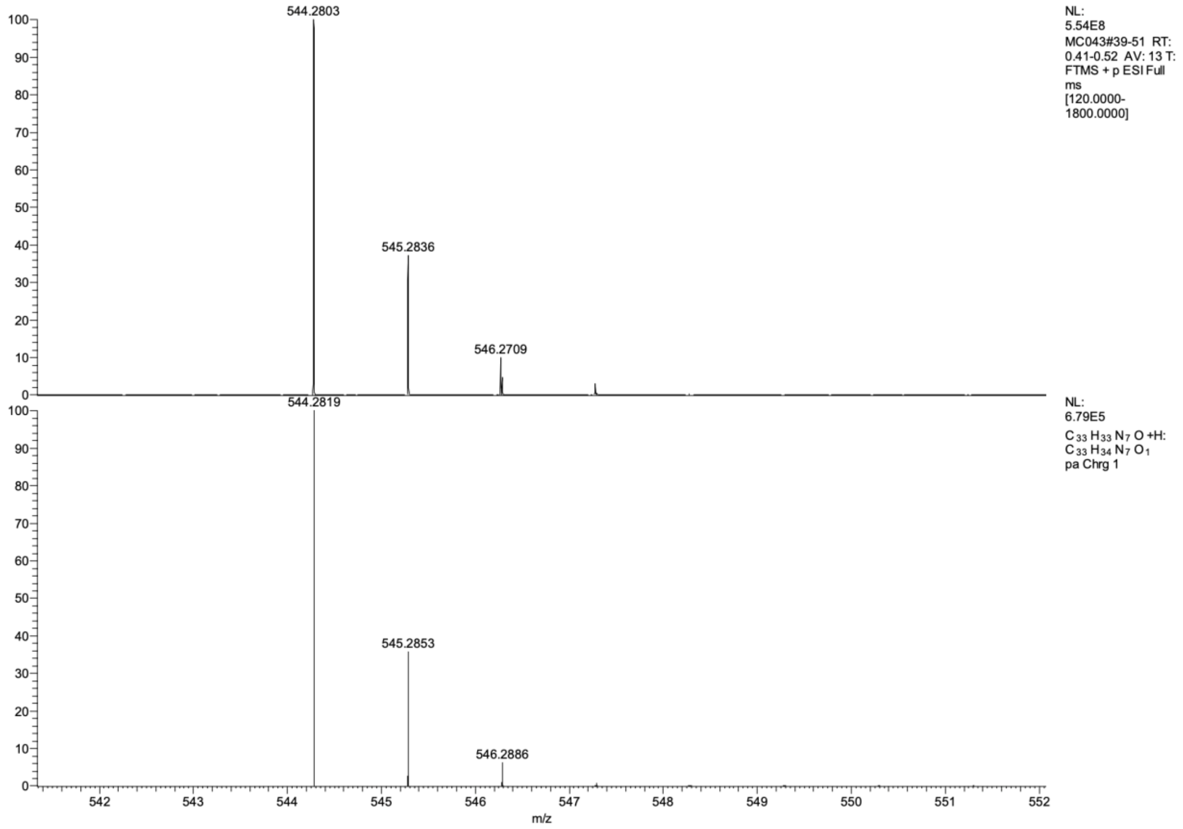
544.2803
545.2836
546.2709
NL:
5.54E8
MC043#39-51 RT:
0.41-0.52 AV: 13 T:
FTMS + p ESI Full
ms
[120.0000-
1800.0000]
544.2819
545.2853
546.2886
NL:
6.79E5
C 33 H 33 N 7 O +H:
C 33 H 34 N 7 O 1
pa Chrg 1
m/z

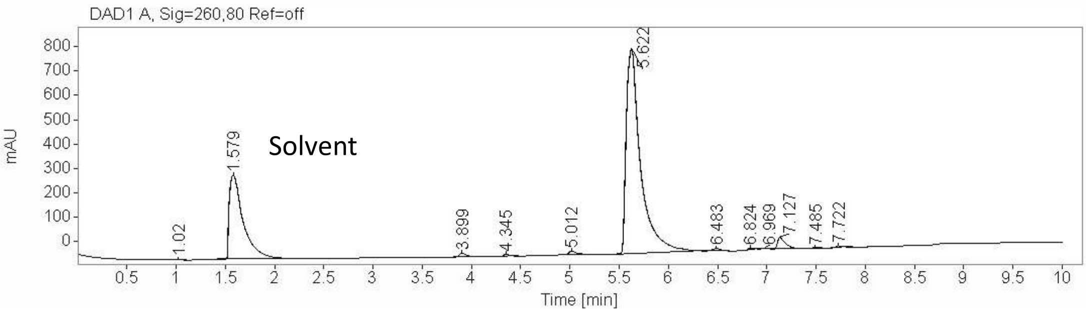
DAD1 A, Sig=260,80 Ref=off
mAU
Solvent
1.02
1.579
3.899
4.345
5.012
5.622
6.483
6.824
6.969
7.127
7.485
7.722
Time [min]

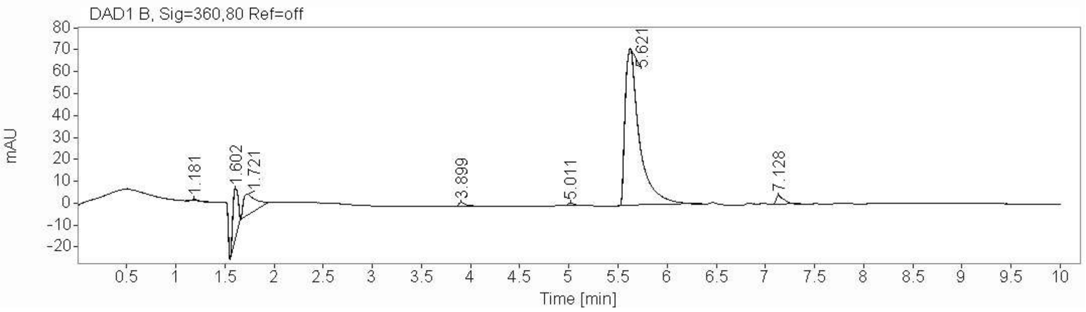
DAD1 B, Sig=360,80 Ref=off
mAU
1.181
1.602
1.721
3.899
5.011
5.621
7.128
Time [min]

Figure S9g: ^{1}H NMR, ^{13}C NMR and HRMS (theoretical result in the bottom and experimental at the top) spectra of **13**

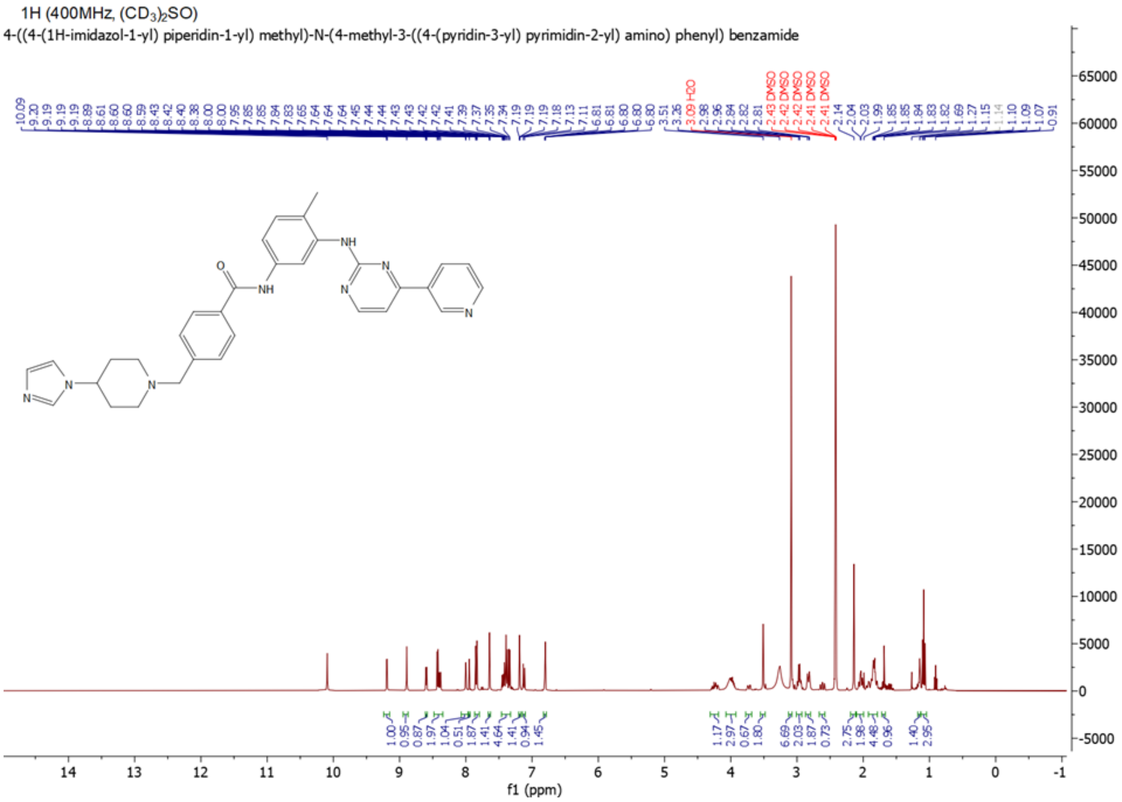

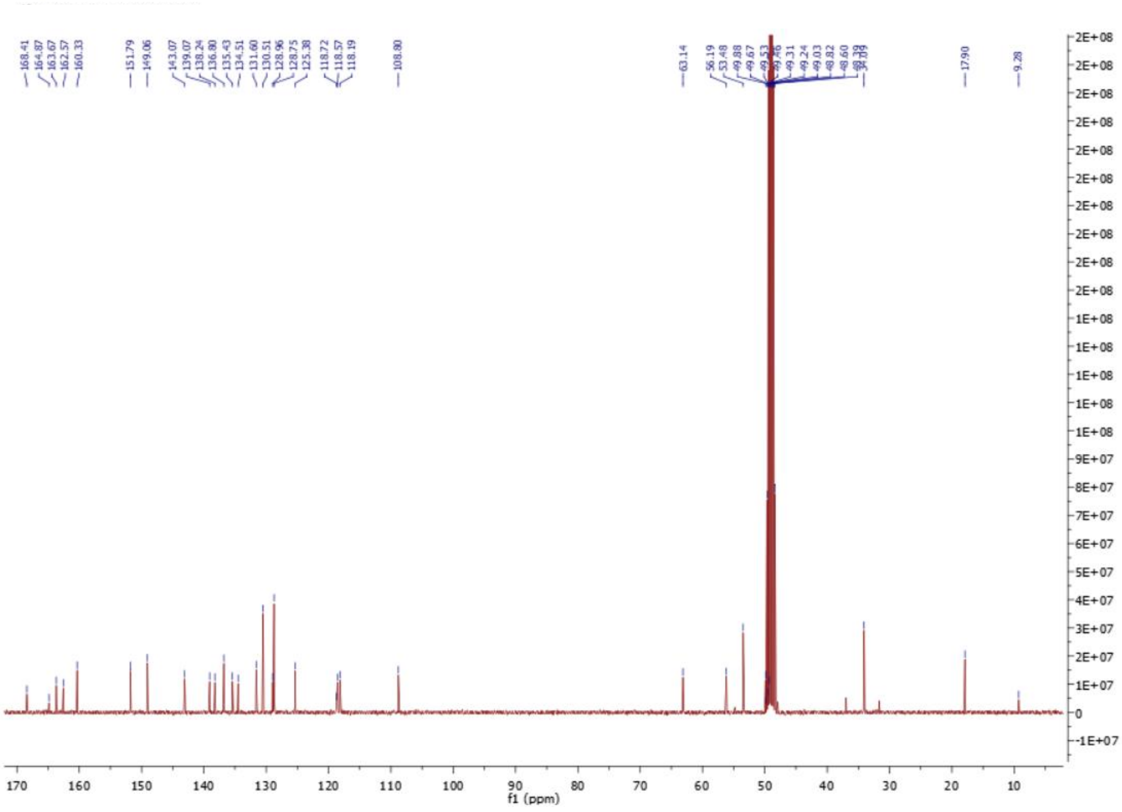

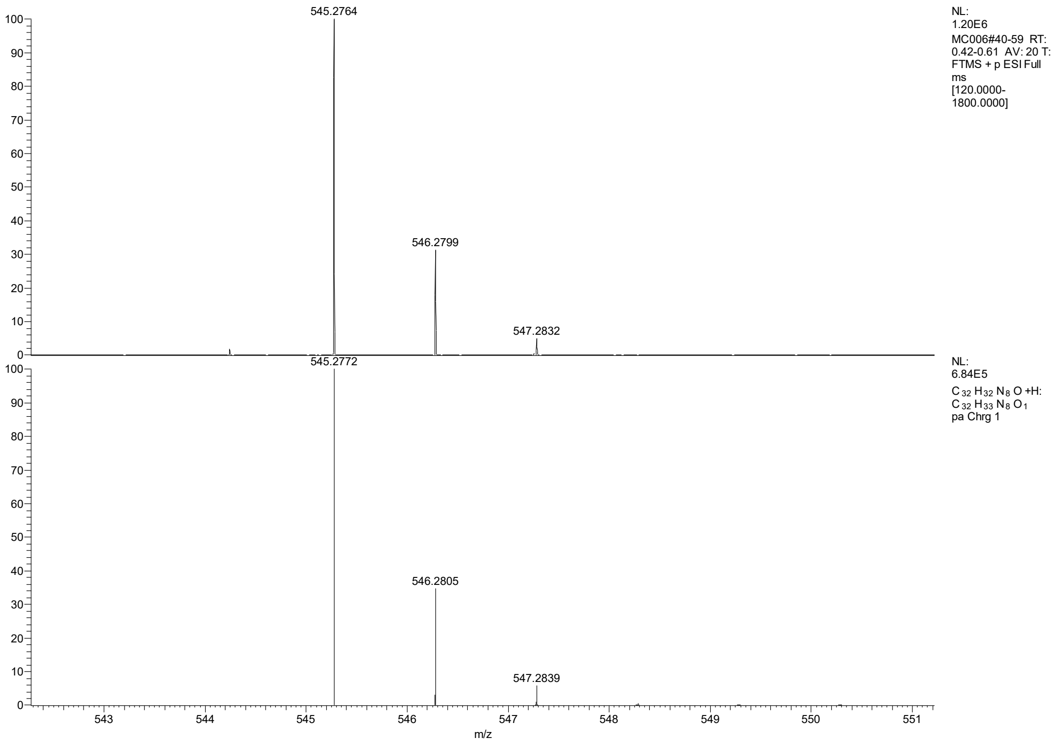

05/04/2022 Orbitrap Q Exactive mass spectrometer 3

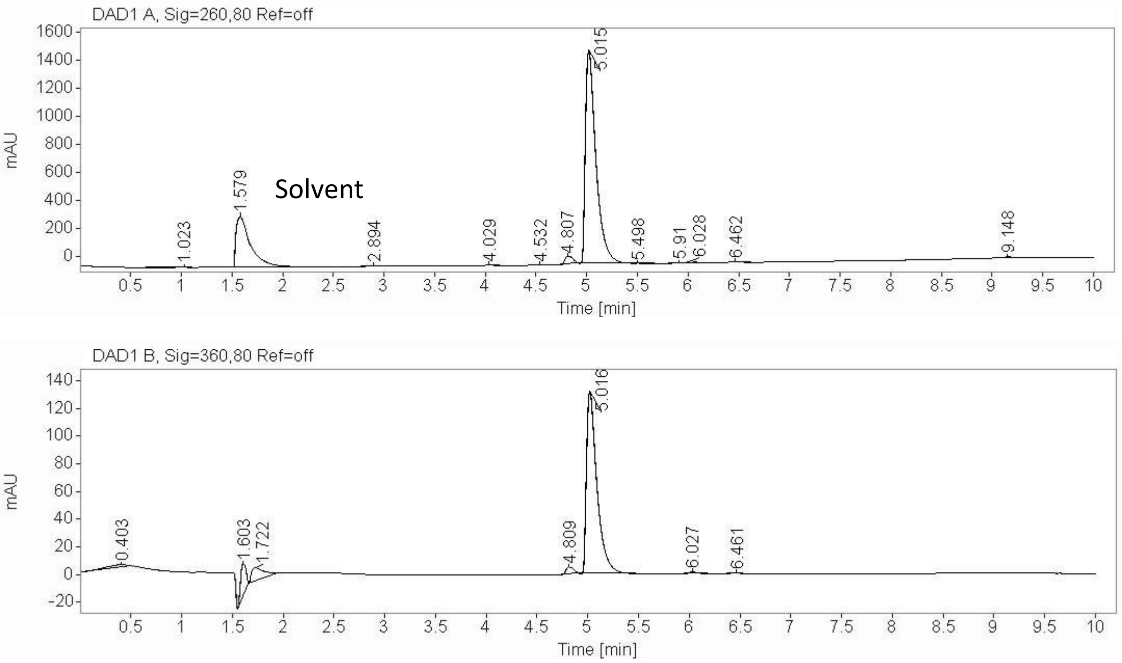

Figure S9h: ^{1}H NMR, ^{13}C NMR and HRMS (theoretical result in the bottom and experimental at the top) spectra of **14**

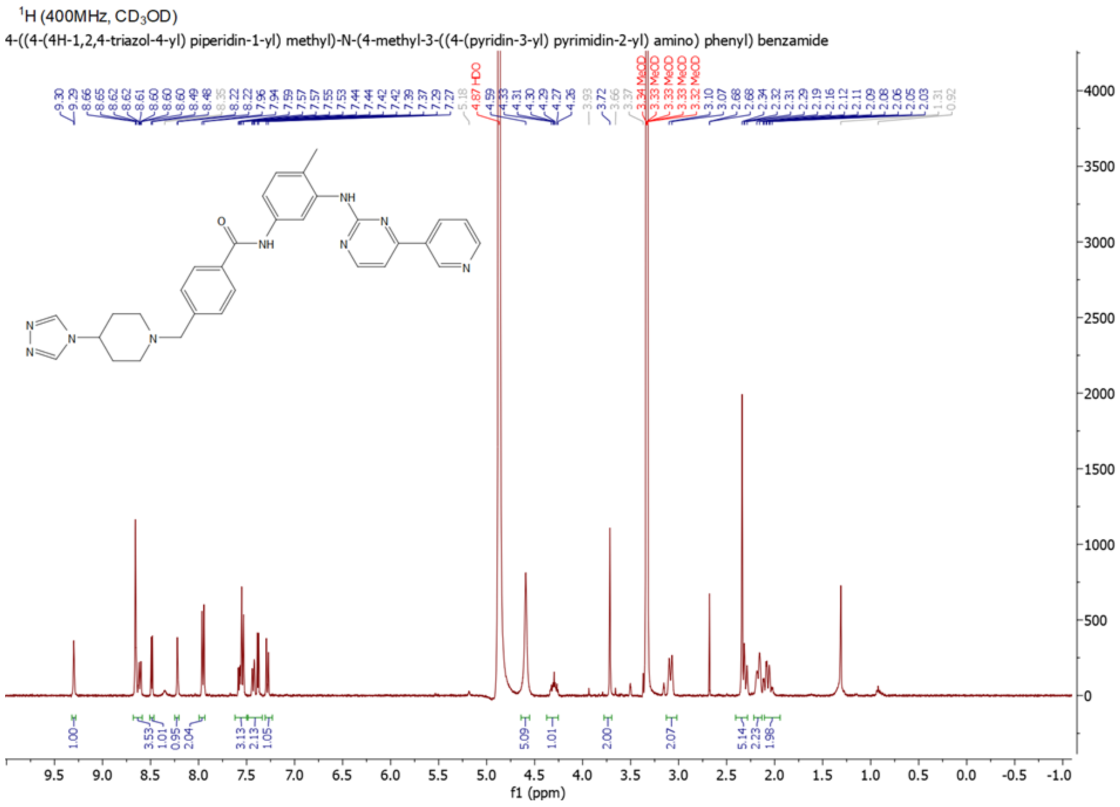

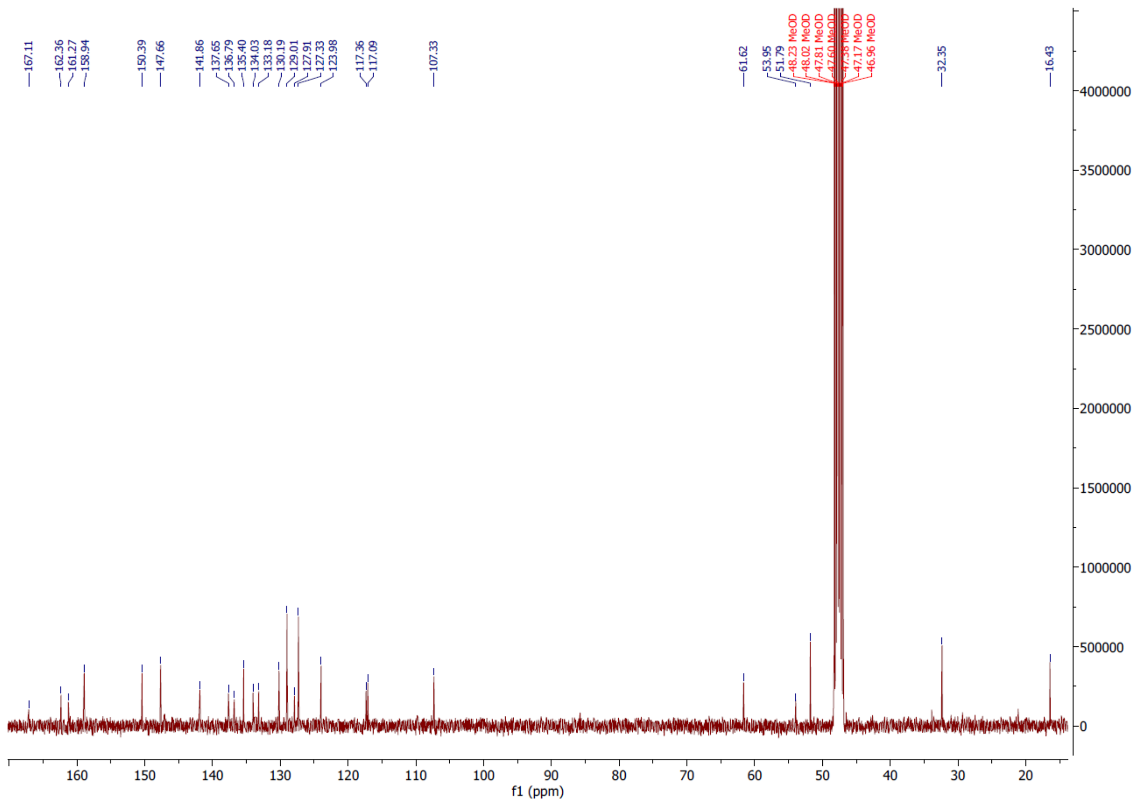

167.11
162.36
161.27
158.94
150.39
147.66
141.86
137.65
136.79
135.40
134.03
133.18
130.19
129.01
127.91
127.33
123.98
117.36
117.09
107.33
61.62
53.95
51.79
48.23 MeOD
48.02 MeOD
47.81 MeOD
47.60 MeOD
47.38 MeOD
47.17 MeOD
46.96 MeOD
32.35
16.43
f1 (ppm)

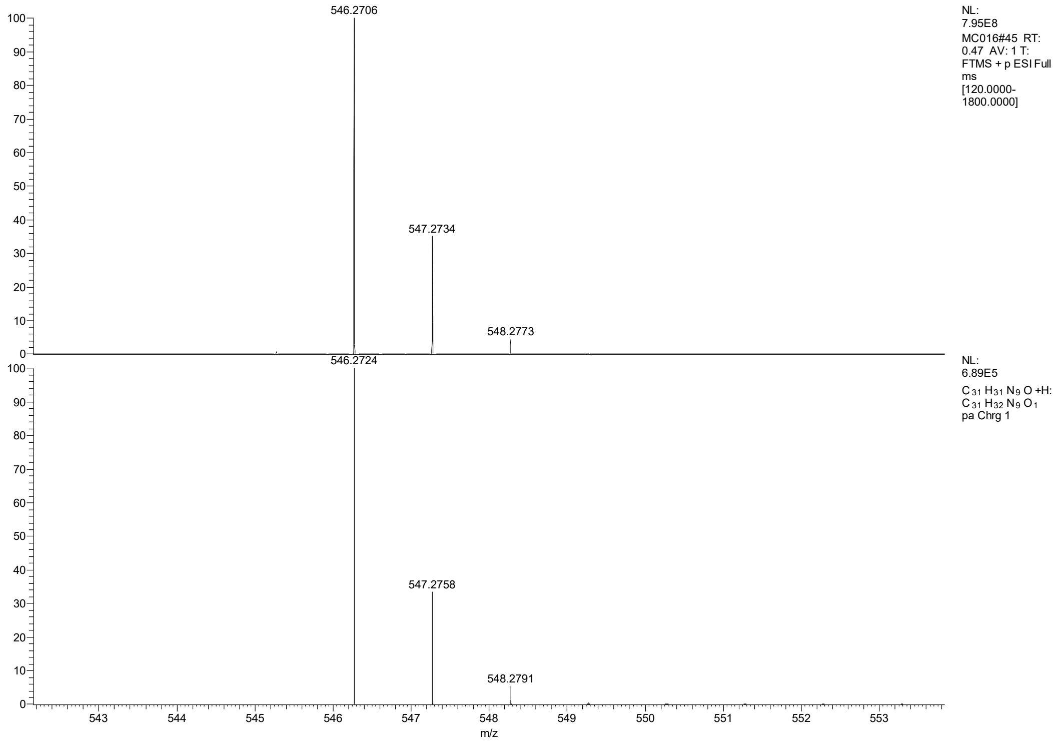

546.2706
547.2734
548.2773
NL:
7.95E8
MC016#45 RT:
0.47 AV: 1 T:
FTMS + p ESI Full
ms
[120.0000-
1800.0000]
546.2724
547.2758
548.2791
NL:
6.89E5
C 31 H31 N9 O +H:
C 31 H32 N9 O1
pa Chrg 1
m/z

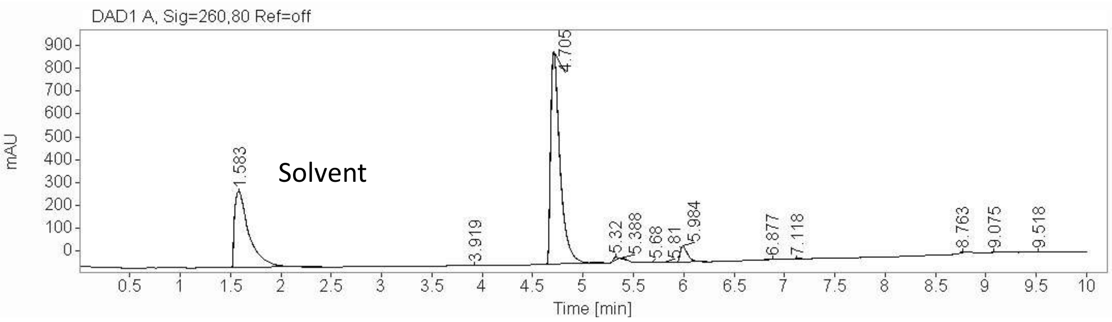

DAD1 A, Sig=260,80 Ref=off
mAU
Solvent
1.583
3.919
4.705
5.32
5.388
5.68
5.81
5.984
6.877
7.118
8.763
9.075
9.518
Time [min]

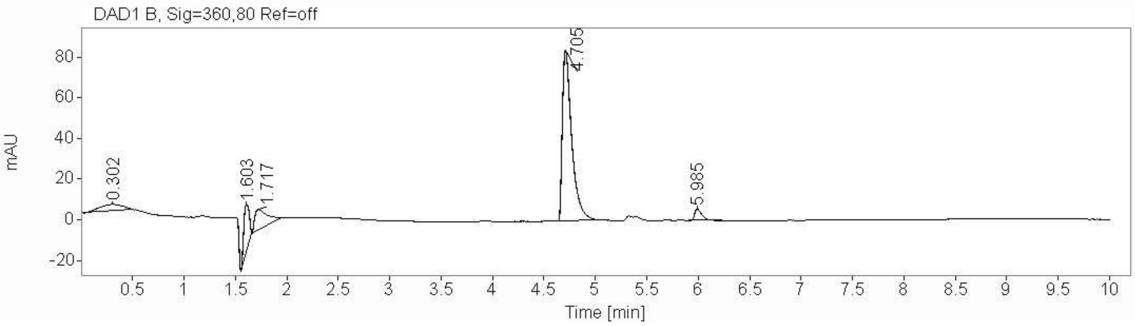

DAD1 B, Sig=360,80 Ref=off
mAU
0.302
1.603
1.717
4.705
5.985
Time [min]

Percentage purity for final compounds as determined by HPLC

Table S3: Table showing percentage purity for final compounds as determined by 10-minute run on HPLC via method B.

**Compound**	Percentage purity
**7**	96.0442
**8**	99.7452
**9**	97.4481
**10**	97.4774
**11**	95.0324
**12**	95.5819
**13**	96.7546
**14**	94.2446
